# Supplementary material for: Evolutionary differentiation of androgen receptor is responsible for sexual characteristic development in a teleost fish
Source: Nat Commun. 2023 Mar 14;14:1428. doi: 10.1038/s41467-023-37026-6 (PMC10014959; doi:10.1038/s41467-023-37026-6)
Supplement: Supplementary file 1 — Supplementary Information [file 41467_2023_37026_MOESM1_ESM.pdf]

Supplementary Information

Evolutionary differentiation of androgen receptor is responsible for sexual characteristic development in a teleost fish

Supplementary Materials and Methods
Supplementary Figures
Supplementary Tables

Supplementary Materials and Methods

Section *in situ* hybridization

Section *in situ* hybridization analysis was performed as described previously[1]. The cDNAs used for preparation of antisense riboprobes of *vasa*, *P450c17*, and *gsdf* were cloned by standard reverse transcription polymerase chain reaction (RT-PCR) procedures using primers shown in Supplementary Table 1.

Targeted gene disruption with TALENs

Genome editing mediated by transcription activator-like effector nucleases (TALENs) was performed as previously reported[2]. Ar proteins contain a N-terminal domain (NTD), DNA binding domain (DBD), and ligand binding domain (LBD) from the amino to carboxyl terminus. The TALEN pairs for *ara* and *arb* were designed to cleave the 1st exon of *ara* and *arb* at the downstream of their ATG start codons, respectively (their binding sequences are shown in Supplementary Fig. 12). Synthesis and purification of capped RNAs from the linealised TALEN expression vectors was performed using the mMessage mMachine SP6 kit (Thermo Fisher Scientific, Waltham, MA, USA) and the RNeasy Mini kit (Qiagen, Hilden, Germany). The pairs of RNA for TALENs were microinjected into fertilized medaka eggs at the 1-cell stage. F_0 founders were crossed with WT fish and then germline-transmitted mutant Fish (F_1) were selected by direct sequencing of the PCR products amplified from the fin clips of the adult fish using the primers, ara-F and ara-R for *ara*, and arb-F and arb-R for *arb* (Supplementary Table 1). The genetic sex of each fish was examined by genomic PCR experiments according to a previous report[3]. The mutant strains were maintained by crossing heterozygous females and males, and the resulting WT and homozygous siblings were used for phenotypic analyses. The *ara* and *arb* double heterozygous males and females ($ara^{+/-}$; $arb^{+/-}$) were obtained by breeding $ara^{-/-}$ males with $arb^{-/-}$ females. The *ara* and *arb* double heterozygous males and females were crossed to obtain males ($ara^{-/-}$; $arb^{+/-}$) and females ($ara^{-/-}$; $arb^{+/-}$). The *ara* and *arb* double homozygous males and females ($ara^{-/-}$; $arb^{-/-}$) were obtained by breeding males ($ara^{-/-}$; $arb^{+/-}$) with females ($ara^{-/-}$; $arb^{+/-}$).

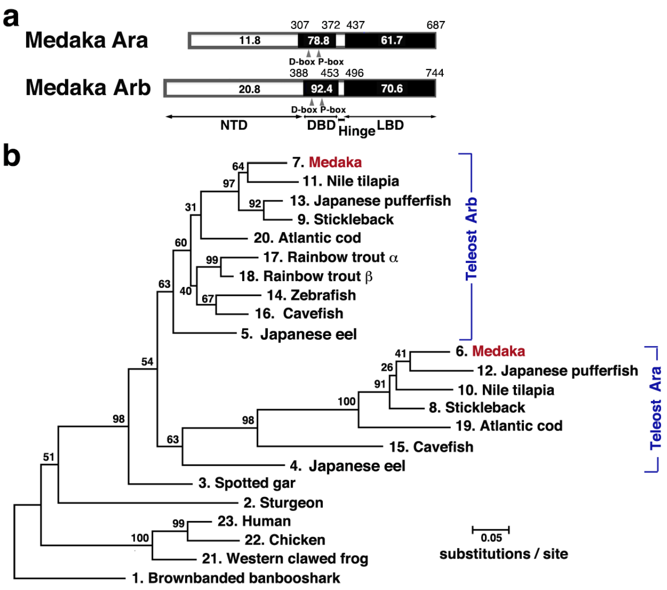

c

	Sequence ID	Annotation
1	AB213019	Brownbanded bambooshark Ar
2	AB213020	Sturgeon (bester) Ar
3	ENSLOCP00000018074	Spotted gar Ar
4	AB023960	Japanese eel Ara
5	AB025361	Japanese eel Arb
6	AB252233	Medaka Ara
7	AB252679	Medaka Arb
8	AB545869	Stickleback Ara
9	AB545870	Stickleback Arb
10	AB045211	Nile tilapia Ara
11	AB045212	Nile tilapia Arb
12	ENSTRUP00000012893	Japanese pufferfish Ara
13	ENSTRUP00000031457	Japanese pufferfish Arb
14	EU708622	Zebrafish Ar
15	ENSAMXP00000013640	Cavefish Ara
16	ENSAMXP00000011713	Cavefish Arb
17	AB012095	Rainbow trout Ara
18	AB012096	Rainbow trout Arb
19	ENSGMOP00000004335	Atlantic cod Ar
20	ENSGMOP00000010954	Atlantic cod Ar
21	ENSXETP00000011091	Western clawed frog Ar
22	AB193190	Chicken Ar
23	NM_000044.2	Human Ar

Supplementary Fig. 1

Phylogenetic analysis of Ar genes

a Structures of medaka Ara (GenBank accession number: AB252233) and Arb (AB252679) proteins. Ar is composed of three major functional domains, a hypervariable N-terminal domain (NTD), a central highly conserved DNA binding domain (DBD) containing two zinc finger motifs, and a COOH-terminal ligand binding domain (LBD). The numbers above each box refer to the position of amino acids in the putative DBD and LBD. The % of identity of deduced amino acid sequences of each domain to human AR (NM_000044.2) is shown in boxes.

b A molecular phylogenetic tree of vertebrate *ar* genes. This tree was estimated with Ar protein sequences using the maximum-likelihood (ML) method combined with JTT substitution model. The Brown-banded bambooshark *ar* gene was used as an outgroup. Support values at nodes are bootstrap probabilities in the ML analysis.

c GenBank and Ensembl accession numbers of the gene sequences used in this analysis and their species names. This figure was modified from Ogino *et al.* 2016[4] by taking a permission to reuse (license Number 5411120388057).

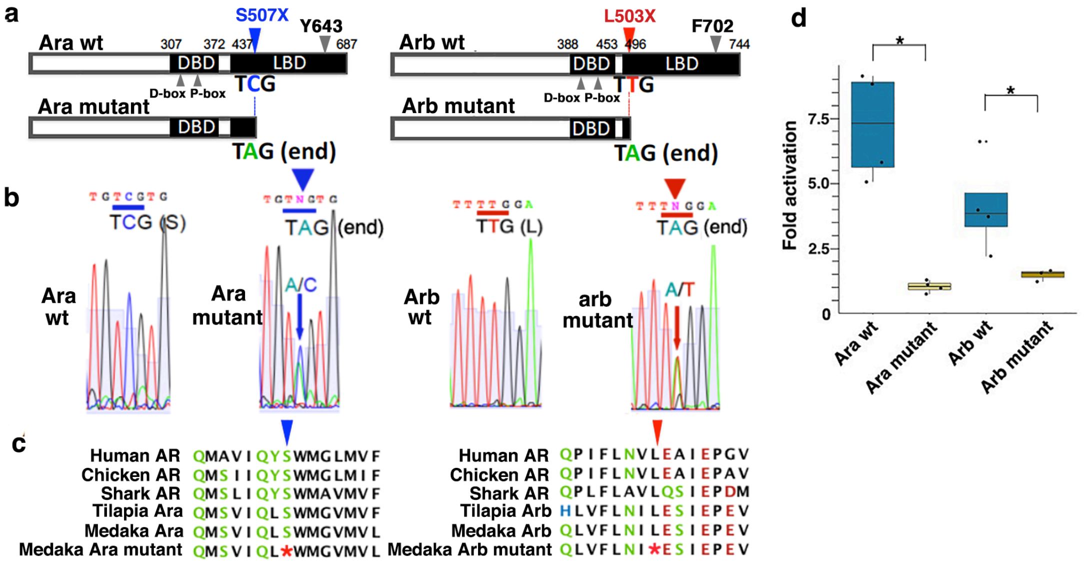

Supplementary Fig. 2

Identification and verification of *ara/arb* TILLING mutant medaka

a Protein structures of medaka Ara and Arb, and positions of the mutations identified in this study. An amino acid in the Arb LBD, F702, is conserved in ancestral-type tetrapod Ar proteins. The corresponding amino acid is replaced with Y634 in Ara, which is responsible for different ligand-dependent transcriptional responses of Ara and Arb *in vitro*[4]. The nonsense mutations in *ara* (S507X) and *arb* (L503X) resulted in expression of the truncated protein products lacking the LBD.

b Nucleotide sequence data of WT and heterozygous mutant (+/-) *ara* and *arb* loci. Nucleotides encoding serine (S) in the WT was altered to a stop codon by cytosine-to-adenine substitution in *ara* (+/-). Nucleotides encoding leucine (L) in WT was altered to a stop codon by a thymine-to-adenine substitution in *arb* (+/-).

c Amino acid sequence alignments of the mutated regions of medaka Ara and Arb with the orthologous regions of human, chicken, shark, tilapia and WT medaka Ars. Arrowheads indicate the premature stop codons (*) in Ara and Arb of the TILLING mutant medaka. Human AR: NM_000044.2; chicken Ar: AB193190; brown-banded bambooshark Ar: AB213019; Nile tilapia Ara: AB045211, Nile tilapia Arb: AB045212 medaka Ara: AB252233; medaka Arb: AB252679.

d Loss of ligand-dependent transactivation activities of the mutant Ara and Arb proteins. COS-7 cells were transfected with an expression plasmid carrying either ara^+, ara^-, arb^+ or arb^- allele-derived cDNA (pCMV-medaka Ara^+, pCMV-medaka Ara^-, pCMV-medaka Arb^+, or pCMV-medaka Arb^-) and a reporter plasmid carrying an

Ar-response element (pGL3 PRE/ARE tk Luc). pRL-SV40 was also co-transfected to use as an internal control to normalise transfection efficiency. Transfected cells were treated with 10^{-8} M 11KT to activate Ar or vehicle alone as a negative control. Ligand-dependent transcriptional activity of Ar is shown as the fold activation relative to the negative control. Data were collected by four independent experiments with means of three technical replicates. The blue, light brown, and dark brown colors indicate WT, the mutant Ara protein, the mutant Arb protein, respectively. Statistical differences were assessed using two-sided Mann-Whitney U test (R version 4.2.0). *$P < 0.05$ (WT vs Ara mutant: $P = 0.02857$; WT vs Arb mutant: $P = 0.02857$). In the box-plots, the center line indicates the median, box limits indicate the upper and lower quartiles, the whiskers indicate 1.5× interquartile range, and the points are outliers. Source data and statistical data are provided as a Source Data file and supplementary table, respectively.

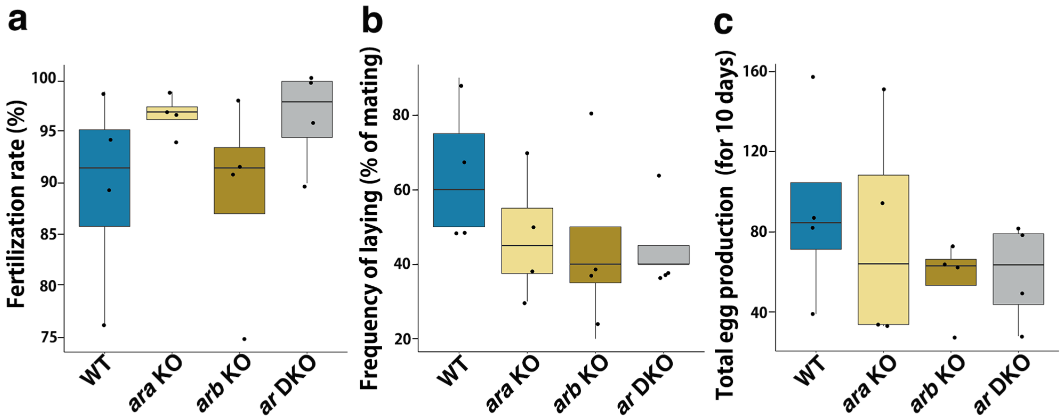

Supplementary Fig. 3

Ar mutant females exhibit normal fecundity

a Fertilisation rates of ovulated oocytes from WT, *ara* KO, *arb* KO, and *ar* DKO females in natural mating with WT males. An average value of mate tests conducted once per day, total ten times, was calculated for each female (n = 4 for WT females, *ara* KO females, *arb* KO females, and *ar* DKO females, respectively).

b Frequency of mating cases in which a female laid eggs within 30 min after initiation of the mating with a WT male. The mate tests were conducted once per day, total ten times.

c Total number of fertilised eggs in 10 days.

Each test female was mated with a different male each day.

The blue, light brown, dark brown, and gray colors indicate WT, *ara* KO, *arb* KO, and

ar DKO, respectively. Statistical differences were assessed using one-way ANOVA followed by two-sided Dunnett's multiple comparison test in the 'glht' function of the R package *multcomp* version 1.4-20. The *ar* genotype did not significantly affect the fecundity of *ar* KO females (one-way ANOVA, **a**: Df= 3, F = 1.317, P = 0.314; **b**: Df= 3, F = 1.072, P = 0.398; **c**: Df= 3, F = 0.65, P = 0.598). The statistical data of two-sided Dunnett's multiple comparison test are provided in supplementary Table 3.

In the box-plots, the center line indicates the median, box limits indicate the upper and lower quartiles, the whiskers indicate 1.5× interquartile range, and the points are outliers. Source data and statistical data are provided as a Source Data file and supplementary Table 3, respectively.

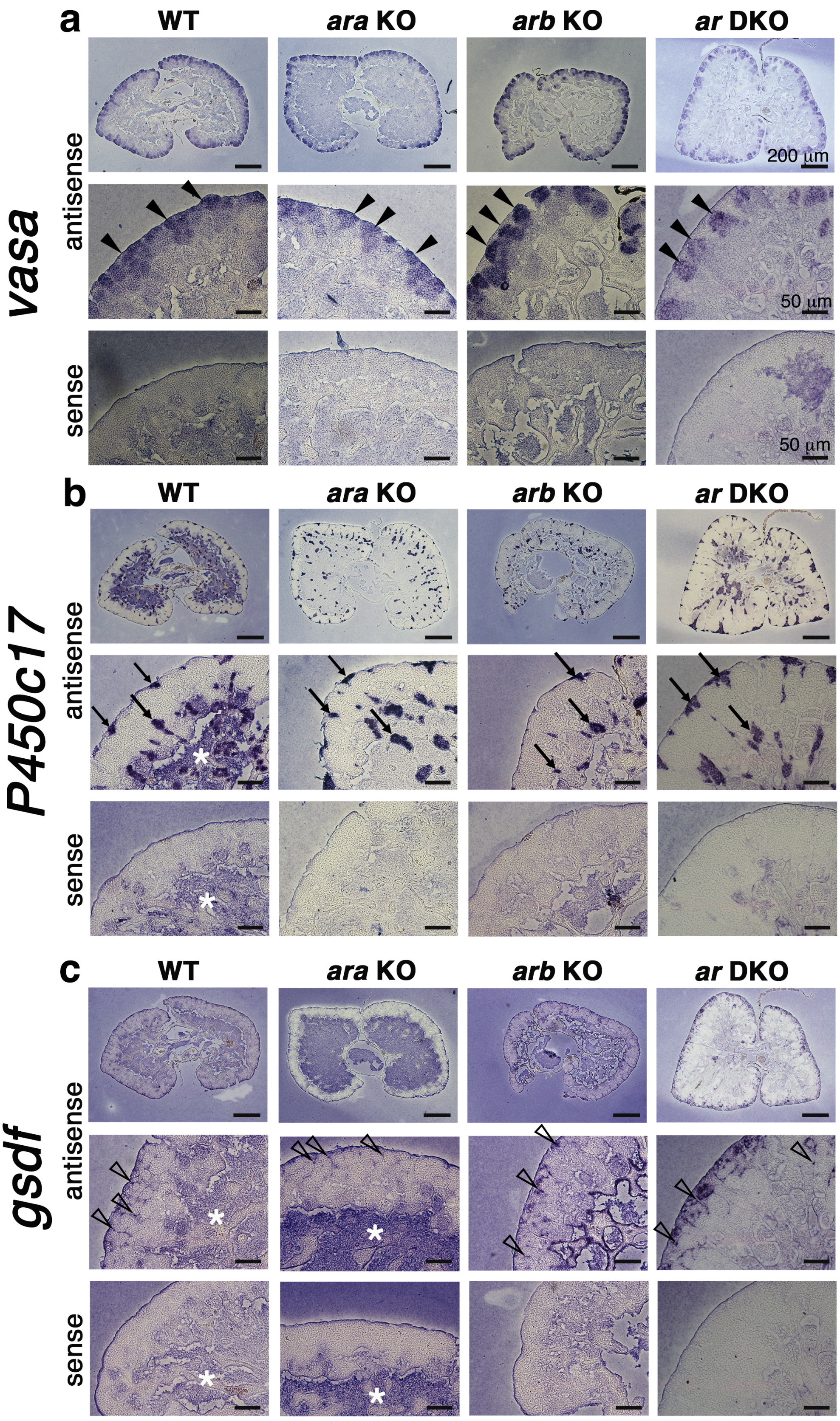

a
WT
ara KO
arb KO
ar DKO
vasa
antisense
sense
200 µm
50 µm
50 µm
b
WT
ara KO
arb KO
ar DKO
P450c17
antisense
sense
c
WT
ara KO
arb KO
ar DKO
gsdf
antisense
sense

Supplementary Fig. 4

***In situ* hybridization for gene expression analysis of testis in WT and *ar* KO strains**

Expression of *vasa* (germ cell marker) (**a**), *P450c17* (Leydig cell marker) (**b**), and *gsdf* (Sertoli cell marker) (**c**) in the testes of WT, *ara* KO, *arb* KO and *ar* DKO males. Hybridization reactions were performed on the cross sections of testes (8 µm thickness). A control section incubated with a sense riboprobe was compared with a consecutive section hybridized with the antisense probe. Closed arrowheads, arrows, and open arrowheads indicate the representative expression signals of *vasa, P450c17*, and *gsdf,* respectively. Images are representative of two experiments. Asterisks indicate background staining signals observed with both antisense and sense probes for the same gene. Scale bars were shown in pictures.

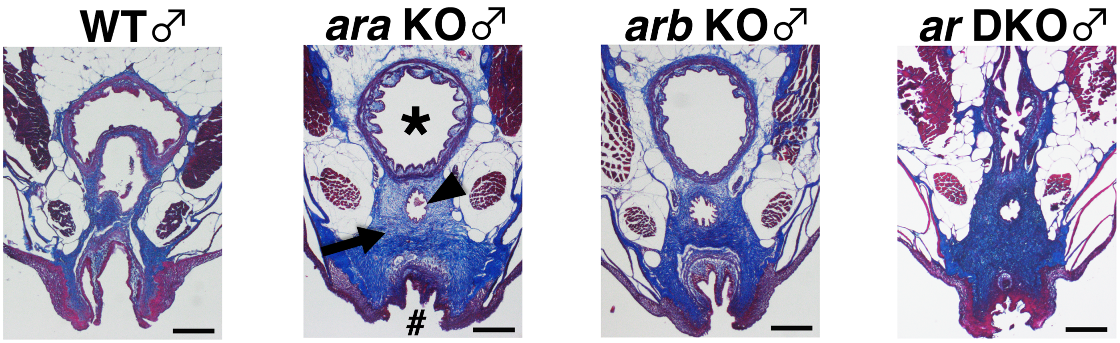

Supplementary Fig. 5

Differentiation of medulla surrounding the sperm duct near the opening of digestive tracts in *ar* KO males

Representative micrographs of Masson/trichrome-stained sections of the urogenital region of WT, *ara* KO, *arb* KO, and *ar* DKO males (n $\geq$ 3 for each genotype). Scale bars represent 200 µm. The urethra, sperm duct, enlarged medulla surrounding the sperm duct, and digestive tract are indicated by *, arrowhead, arrow, and #, respectively.

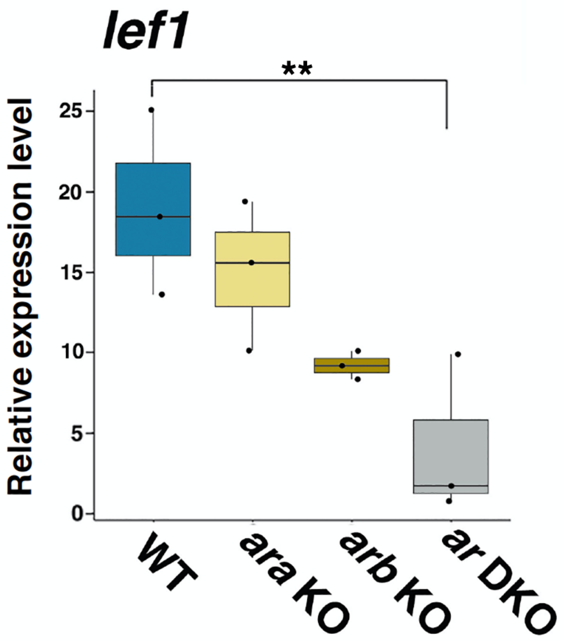

Supplementary Fig. 6

Reduced expression of *lef1* gene in the anal fin of *ar* DKO males.

The graph showed expression level of *lef1* relative to *rpl7* in a posterior half of the anal fin of WT, *ara* KO, *arb* KO, and *ar* DKO medaka (n = 3 for each genotype). The *ar* genotype significantly influenced the *lef1* expression (one-way ANOVA, Df= 3, F = 6.355, P = 0.0164). The two-sided Dunnett's multiple comparison test in the 'glht' function of the R package *multcomp* version 1.4-20 indicated that the *lef1* expression was significantly decreased in the *ar* DKO males (estimate ± s.e. = -14.917 ± 3.671, t = -4.062, P = 0.00931). **P < 0.01. The blue, light brown, dark brown, and gray colors indicate WT, *ara* KO, *arb* KO, and *ar* DKO, respectively. In the box-plots, the center line indicates the median, box limits indicate the upper and lower quartiles, the whiskers indicate 1.5× interquartile range, and the points are outliers. Source data and the other statistical data are provided as a Source Data file and supplementary Table 3, respectively.

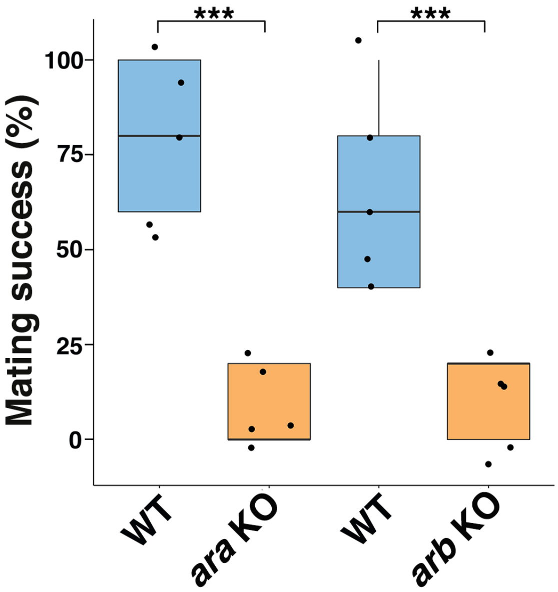

genotype	mating success		total
	+	-	
WT	20	2	22
ara KO	2	20	22
total	22	22	44

genotype	mating success		total
	+	-	
WT	16	3	19
arb KO	3	16	19
total	19	19	38

Supplementary Fig. 7

Reduced reproductive success of *ara* KO males and *arb* KO males compared with WT males

Percentage of mating success cases of WT, *ara* KO, and *arb* KO males in the mate choice tests. The mate choice test was performed using a round-robin tournament with five males.

The two-sided chi-squared ($\chi 2$) test of independence in R version 4.2.0 with the package *lme4* version 1.1.30 was used to assess whether the mutation had a significant effect on mating success (*ara* KO vs WT: $\chi^2 = 23.458$, df = 1, $P = 1.277e\text{-}06$; *arb* KO vs WT: $\chi^2 = 12.224$, df = 1, $P = 0.0004718$, $^{***}P < 0.001$). The blue and light brown colors indicate WT and *ar* KO, respectively. In the box-plots, the center line indicates the median, box limits indicate the upper and lower quartiles, the whiskers indicate 1.5× interquartile range, and the points are outliers. Source data and statistical data are provided as a Source Data file and supplementary table 3, respectively.

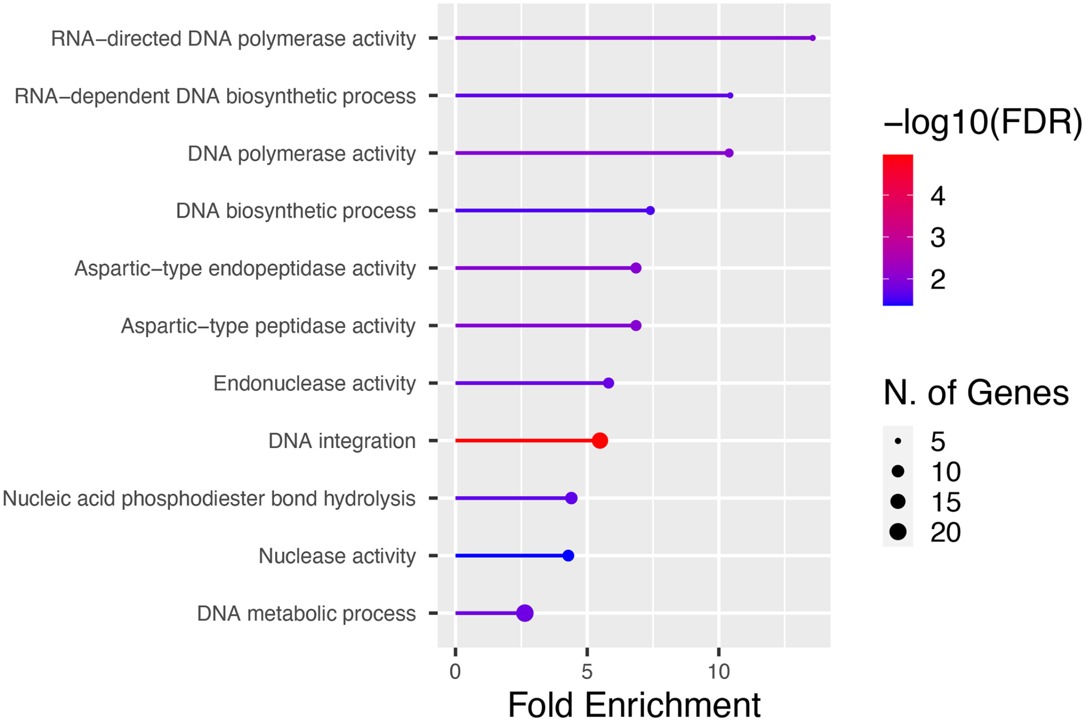

Supplementary Fig. 8

Gene ontology (GO) enrichment analysis of the differentially expressed genes between WT males and *ar* DKO males

Significantly enriched pathways (one-sided hypergeometric test followed by false discovery rate (FDR) correction; FDR cutoff < 0.05) are shown. Colours indicate FDR values. Each dot size represents the number of genes in each pathway, including differentially expressed genes.

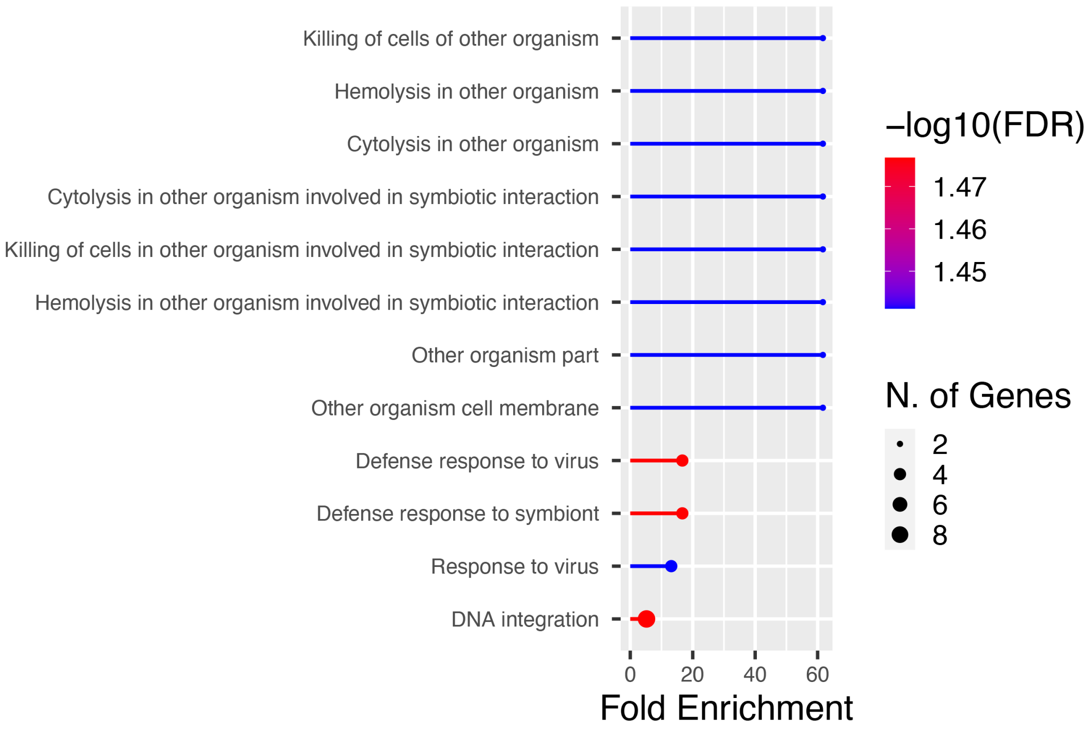

Supplementary Fig. 9

Gene ontology (GO) enrichment analysis of the differentially expressed genes between WT males and *ara* KO males

Significantly enriched pathways (one-sided hypergeometric test followed by false discovery rate (FDR) correction; FDR cutoff < 0.05) are shown. Colours indicate FDR values. Each dot size represents the number of genes in each pathway, including differentially expressed genes.

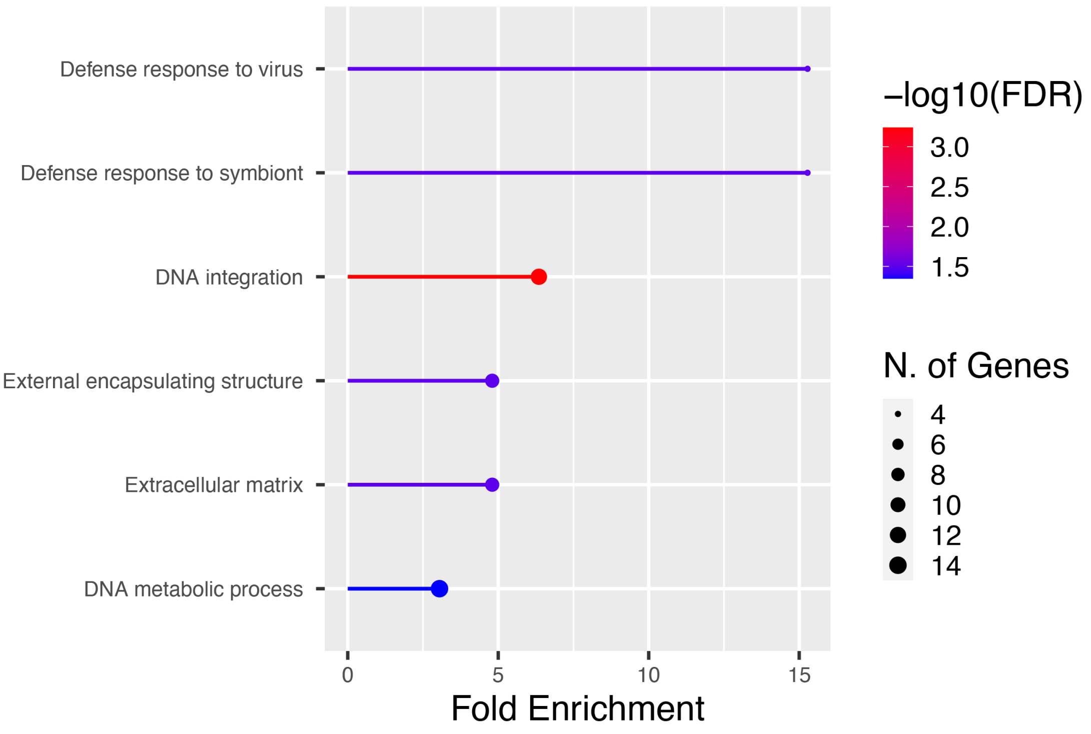

Supplementary Fig. 10

Gene ontology (GO) enrichment analysis of the differentially expressed genes between WT males and *arb* KO males

Significantly enriched pathways (one-sided hypergeometric test followed by false discovery rate (FDR) correction; FDR cutoff < 0.05) are shown. Colours indicate FDR values. Each dot size represents the number of genes in each pathway, including differentially expressed genes.

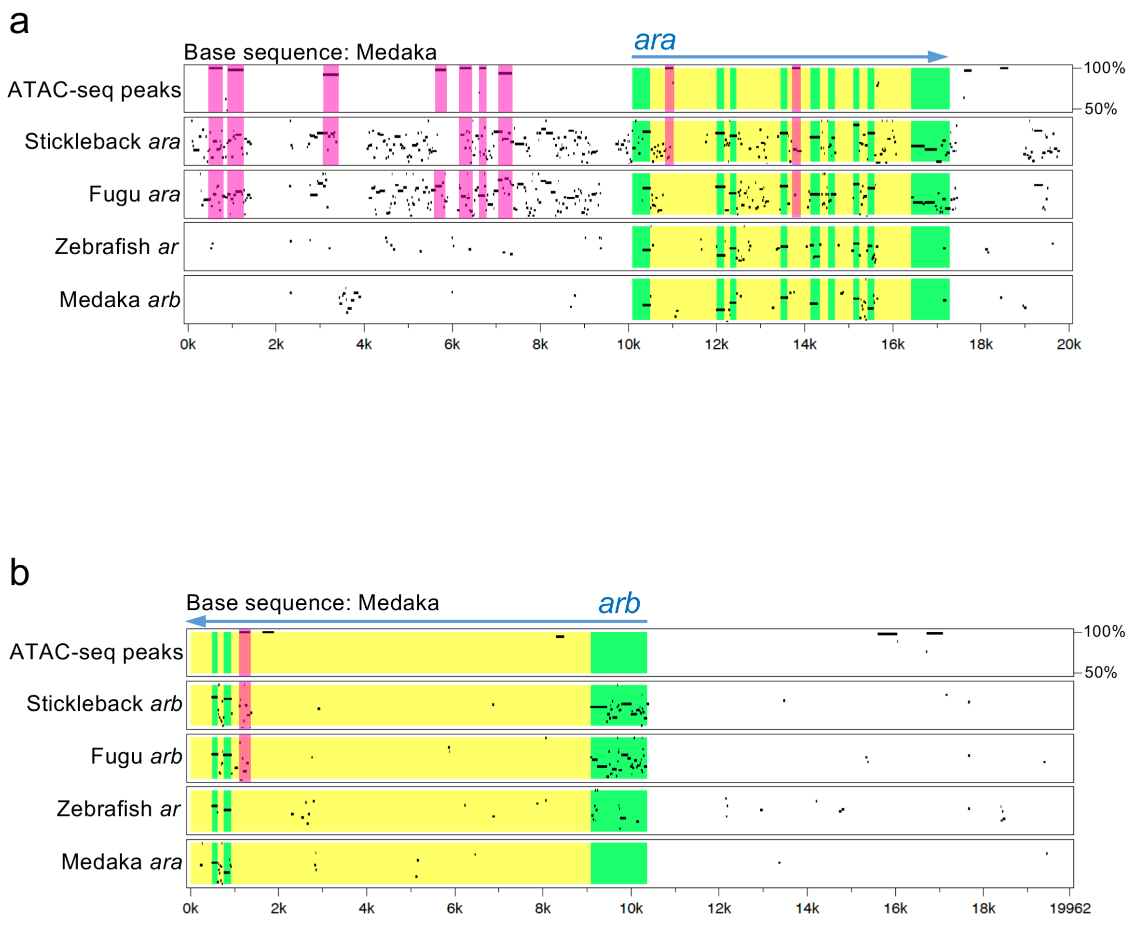

Supplementary Fig. 11

Comparison of *cis*-regulatory sequences of *ar* genes in teleost

a A 20-kb medaka genomic sequence corresponding to *ara* gene and the 5' upstream and 3' downstream regions (oryLat2, chr10: 18,344,871－18,364,872) was aligned with its orthologous sequences from stickleback (gasAcu1, chrIV: 15,180,906－15,200,410), fugu (fr3, chr14: 7,895,689－7,913,481), zebrafish (danRer10, chr5: 34,924,025－35,123,662), a medaka genomic sequence corresponding to *arb* gene and the 5' upstream and 3' downstream flanking regions (ASM223467v1, chr14: 16,689,824－16,772,825), and non-coding ATAC-seq peak sequences associated with the medaka *ara* gene (Li, Y. et al., 2020[5]; http://tulab.genetics.ac.cn/medaka_omics/) using MultiPipMaker web version (http://pipmaker.bx.psu.edu/pipmaker/) to generate a percent identity plot (pip) view[6]. A blue arrow indicates *ara* with its transcriptional orientation. Green and yellow shadings indicate *ara* exons and introns, respectively. Part of the ATAC-seq peak regions is conserved in the *ara* genome sequences of

medaka, stickleback, and/or fugu (shaded in magenta) but not in the zebrafish *ar* or medaka *arb* genome sequences.

b An approximately 20-kb genomic sequence corresponding to the first, second, and third exons of *arb* gene and the 5' upstream region (oryLat2, chr14:17,246,771－17,266,732) was aligned with its orthologous sequences from stickleback (gasAcu1, chrVII:17,122,259－17,143,909), fugu (fTakRub1.2, chr15:12,602,150－12,632,151), zebrafish (danRer10, chr5: 34,924,025－35,123,662), a medaka genomic sequence corresponding to *ara* gene and the 5' upstream region (ASM223467v1, chr10: 22,174,238－22,194,238), and non-coding ATAC-seq peak sequences associated with the medaka *arb* gene (Li, Y. et al., 2020[5]; http://tulab.genetics.ac.cn/medaka_omics/) using MultiPipMaker. Note that the ATAC-seq peaks associated with the medaka *arb* gene localize only within this 20-kb region. A blue arrow indicates *arb* with its transcriptional orientation. Green and yellow shadings indicate *arb* exons and introns, respectively. Only one peak region of ATAC-seq is conserved in the *arb* genome sequences of medaka, stickleback, and fugu (shaded in magenta) but not in the zebrafish *ar* or medaka *ara* genome sequences. These alignments suggest that *cis*-regulatory mechanisms of *ara* and *arb*, respectively, are partially conserved in the teleost species that retain both *ar* ohnologs, but not in the species that lack one or the other, and are not conserved between *ara* and *arb*. The genome sequences used here were downloaded from the UCSC Genome Browser and Ensembl Genome Browser[7,8].

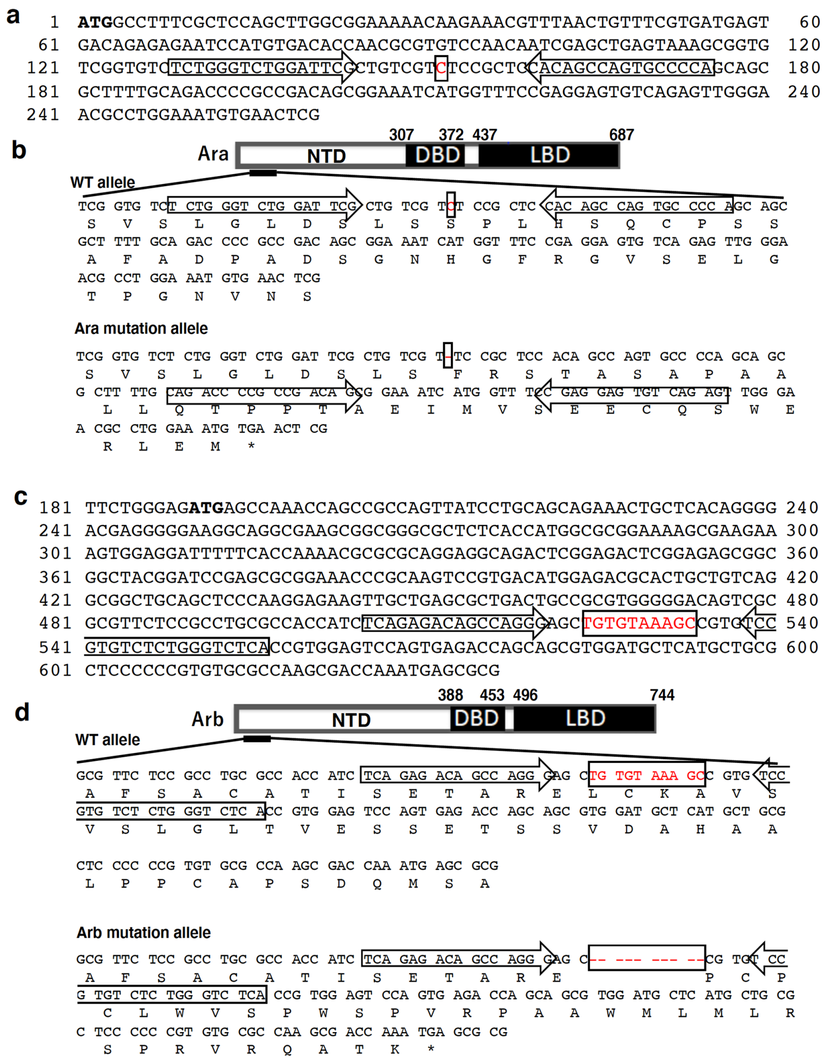

Supplementary Fig. 12

Design of transcription activator-like effector nucleases (TALENs) and frame-shift mutations in the *ara* and *arb* genes

a, c Positions of indels generated in *ar* TALEN KO fish. First ATG sequences in the 1st exon of *ara* or *arb* are in bold characters. Arrows indicate target sequences of the TALEN used in this study. Deleted nucleotides in the *ara* KO and *arb* KO fish are boxed.

b, d Alignments of WT and mutant DNA sequences with their encoding amino acid sequences. The indels introduced by the TALENs are expected to cause frameshifts and generate premature stop codons in the Ara and Arb NTDs. Numbers on the graphical illustration of the functional domains of each Ar protein indicate the start and end residual positions of the DBD and LBD.

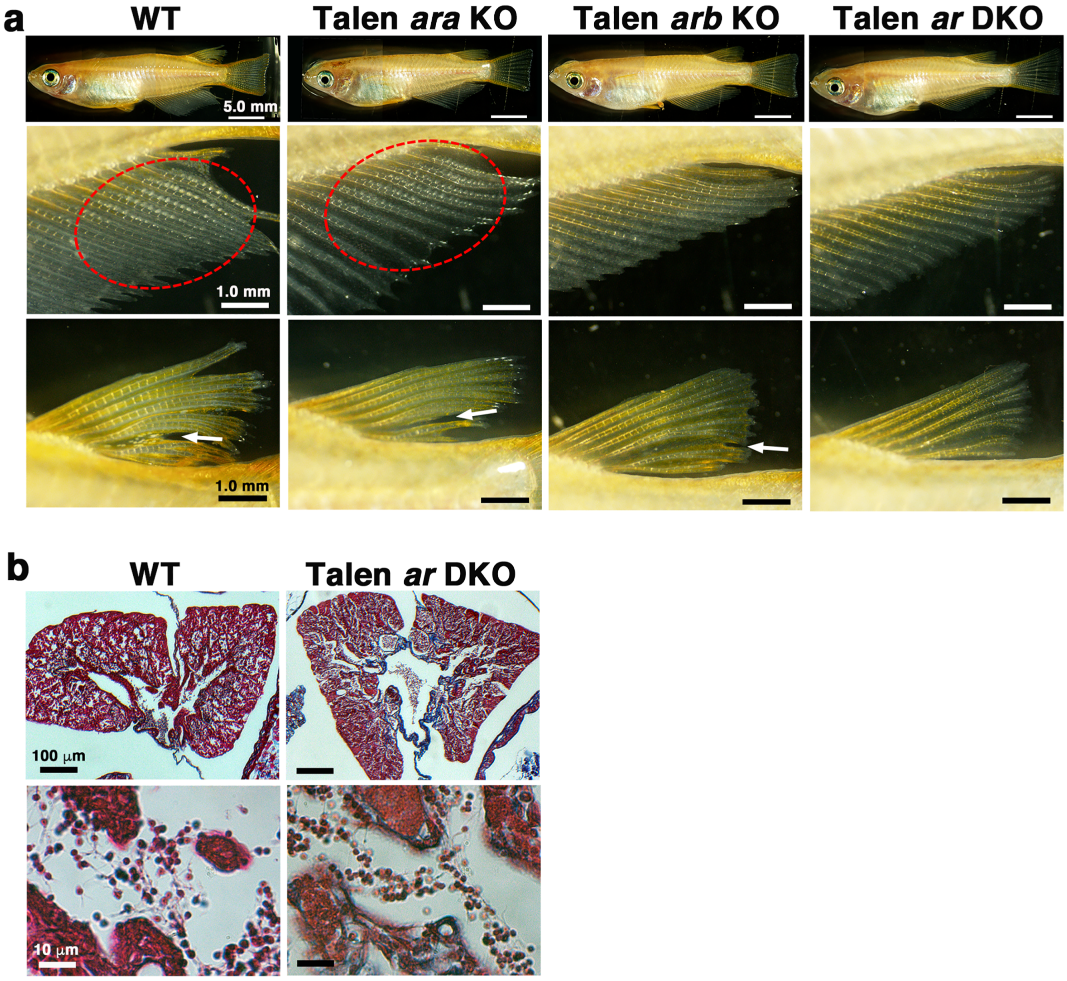

Supplementary Fig. 13

Fin and testicular phenotypes in *ar* TALEN KO medaka

a Representative picture of the whole body and higher magnification pictures of the anal fin and dorsal fin of a WT male, *ara* KO male, *arb* KO male, and *ar* DKO male. The papillary processes developments were marked by red dotted circles. Arrow indicates fork in the dorsal fin.

b Representative micrographs of Masson/trichrome-stained sections of adult gonads of WT males and *ar* DKO males (n $\geqq$ 3 for each genotype). The sexually matured (4 months old) males were utilized for this analysis.

Supplementary Fig. 14

a Chromatograms of 11KT in a standard and testicular extracts (Analyte).

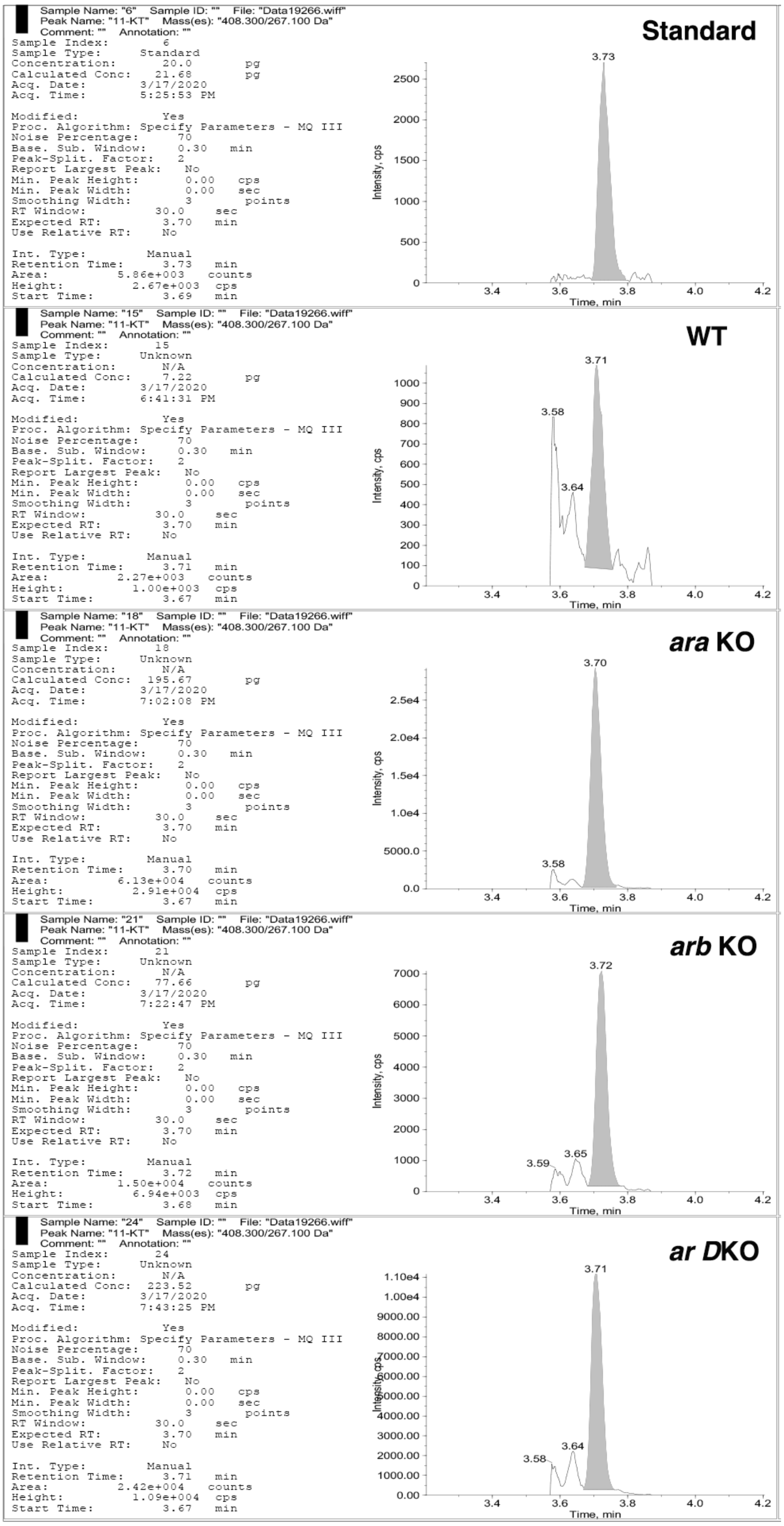

b Chromatograms of 11KT in a standard and testicular extracts (Internal standard). 11-KT-d3 was used an internal standard to precisely scale and measure analyte concentration in LC-MS/MS sample analysis.

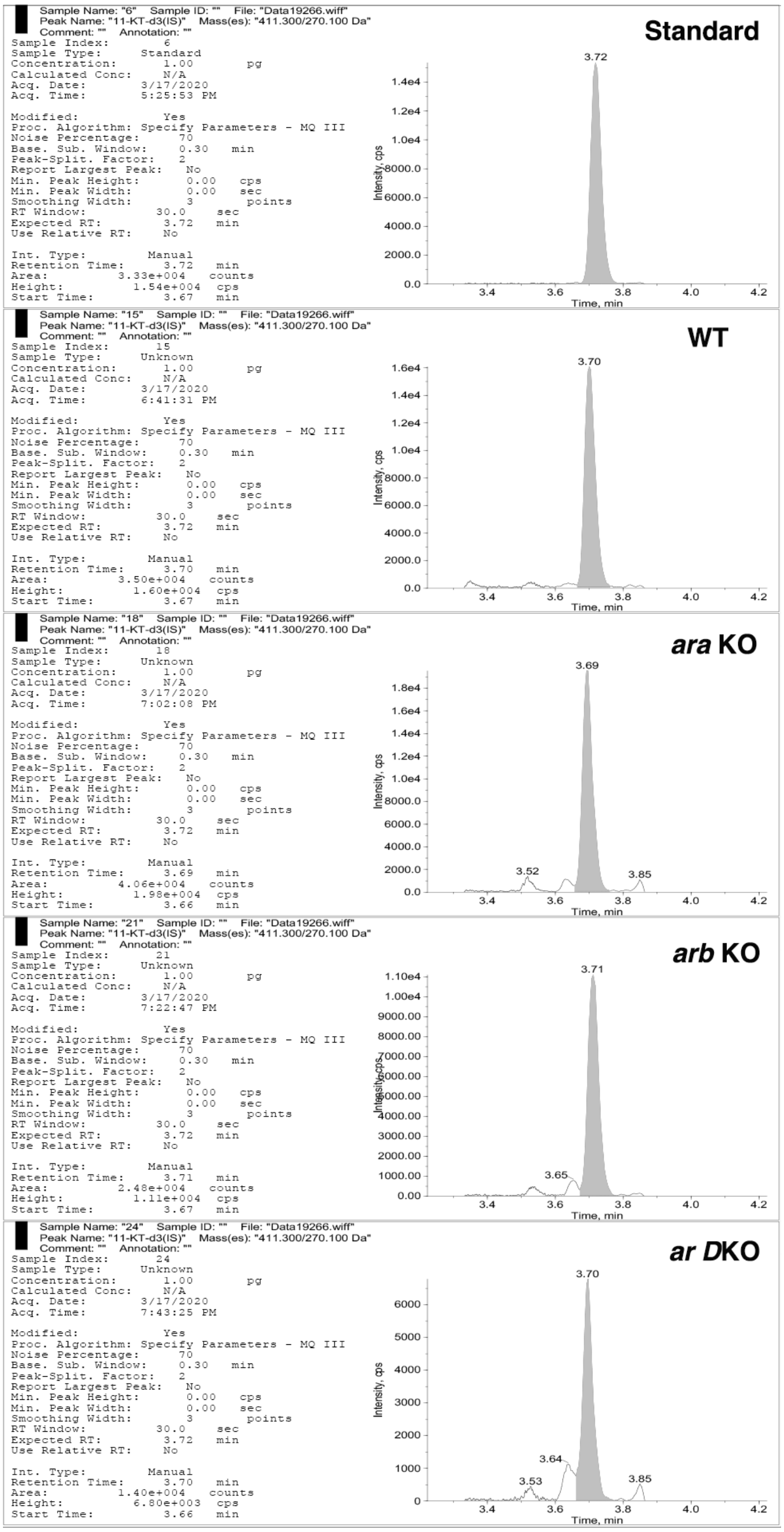

c Chromatograms of T in a standard and testicular extracts (Analyte).

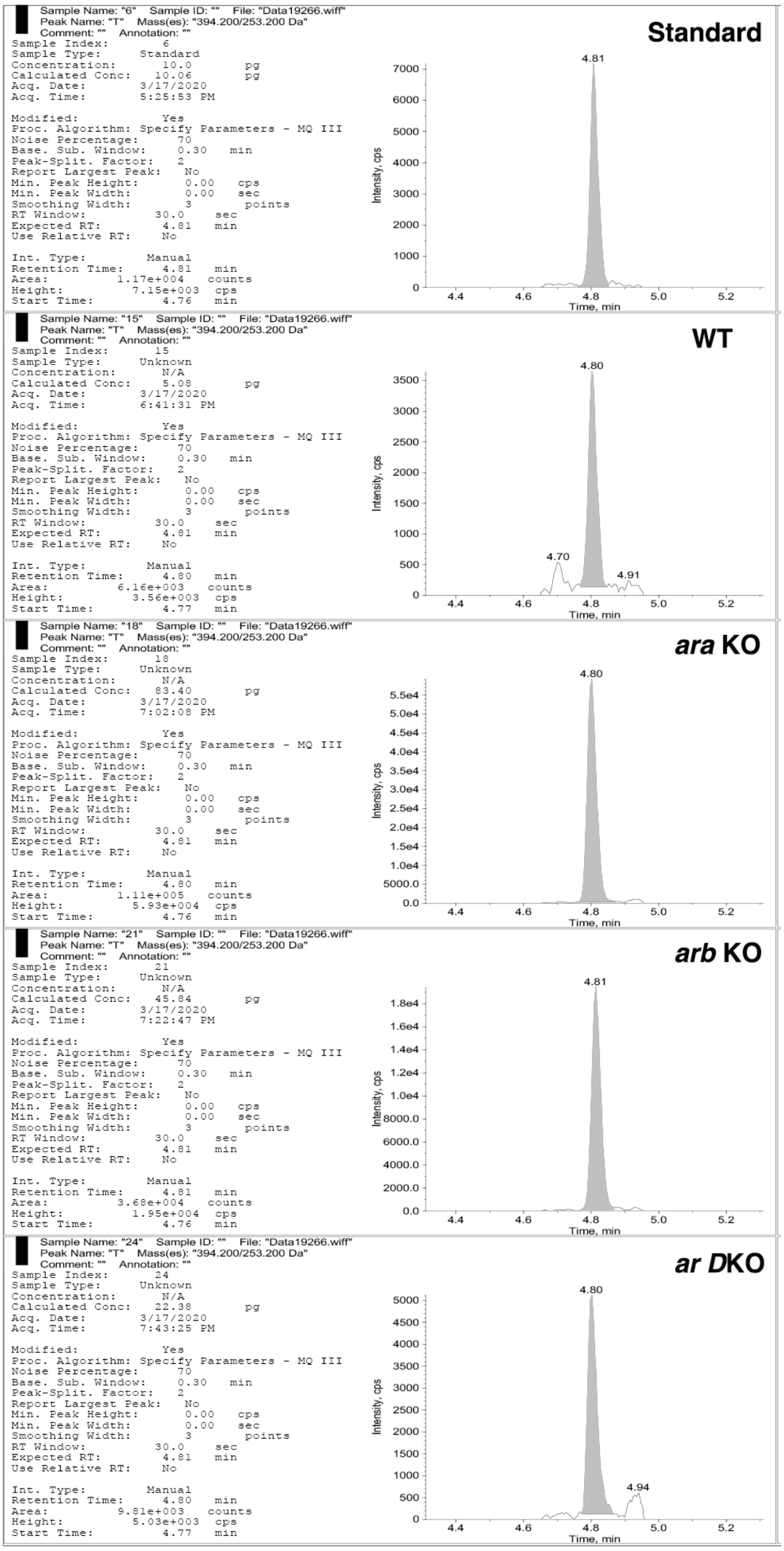

d Chromatograms of T in a standard and testicular extracts (Internal standard). T-13C3 was used an internal standard to precisely scale and measure analyte concentration in LC-MS/MS sample analysis.

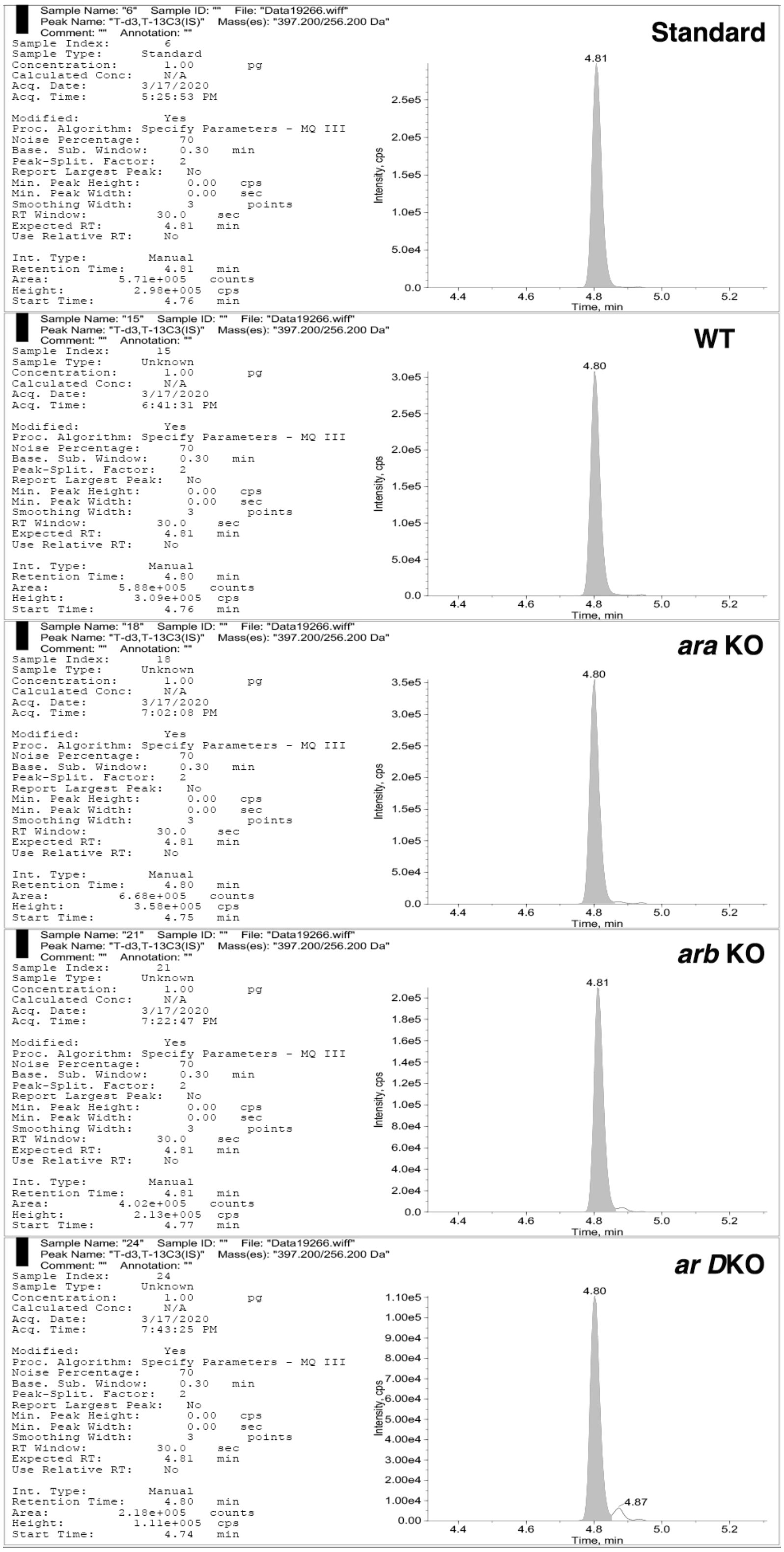

Supplementary Fig. 15

a Chromatograms of 11KT in a standard and brain extracts (Analyte).

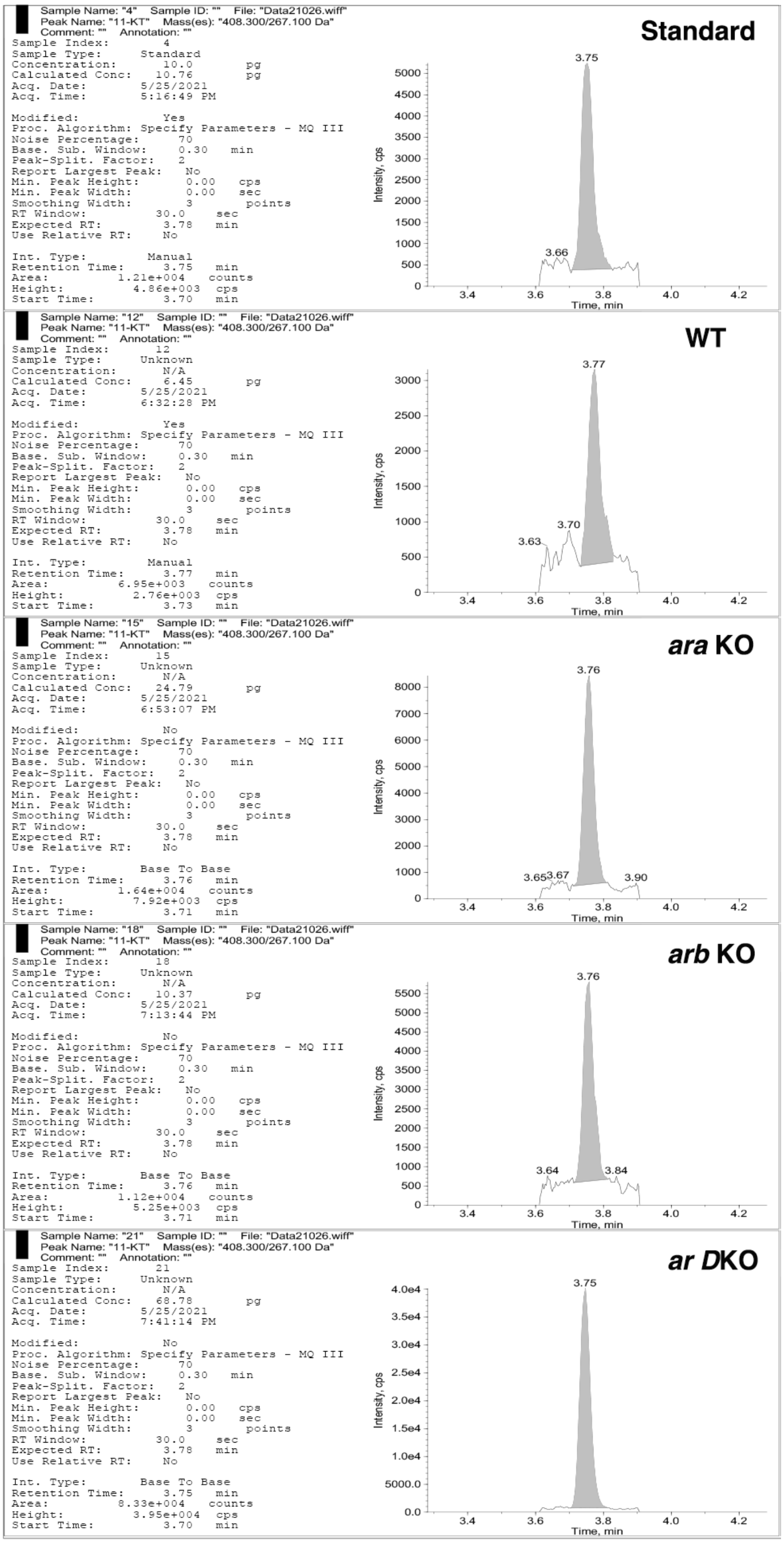

b Chromatograms of 11KT in a standard and brain extracts (Internal standard). 11-KT-d3 was used an internal standard to precisely scale and measure analyte concentration in LC-MS/MS sample analysis.

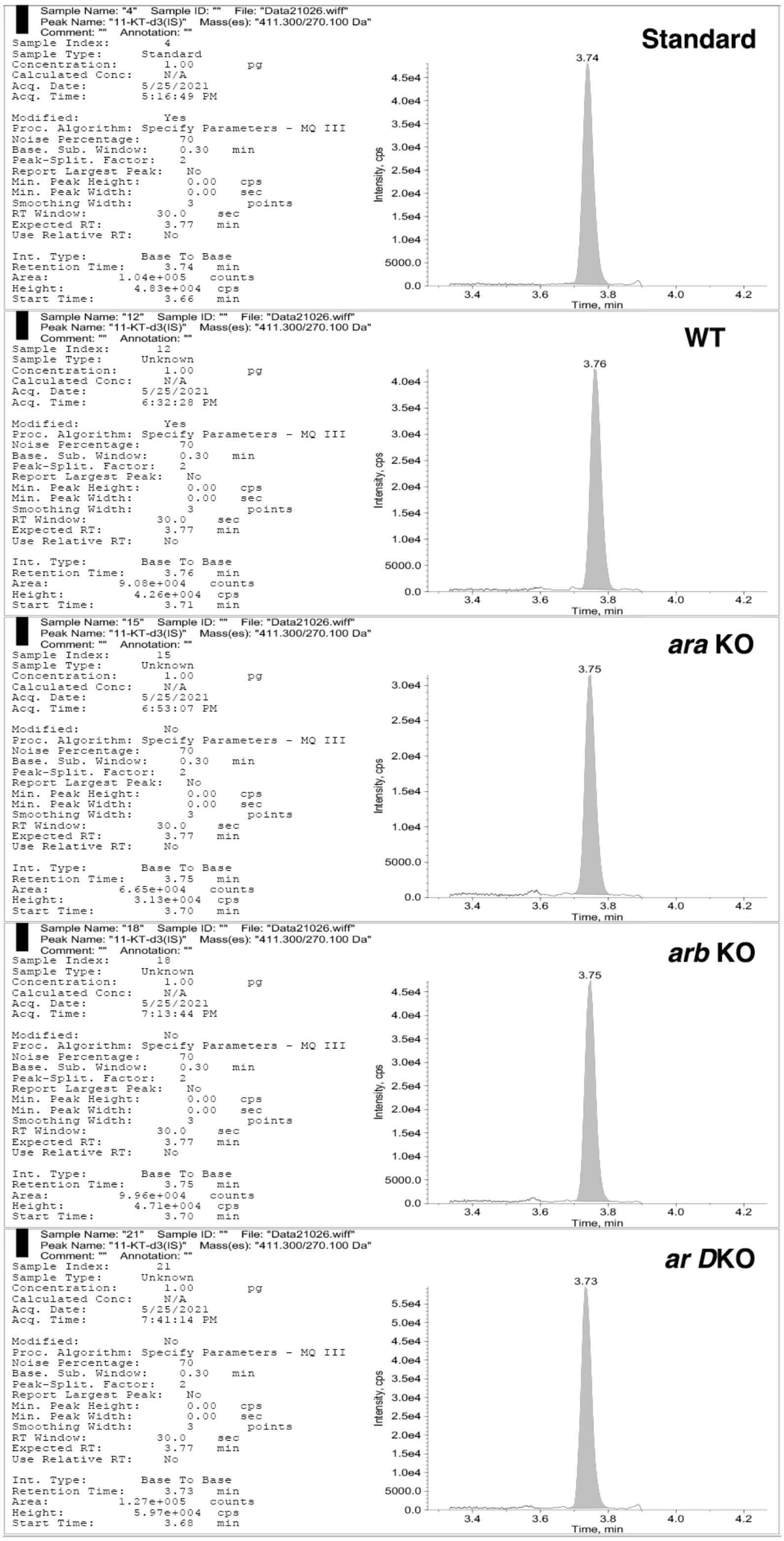

c Chromatograms of T in a standard and brain extracts (Analyte).

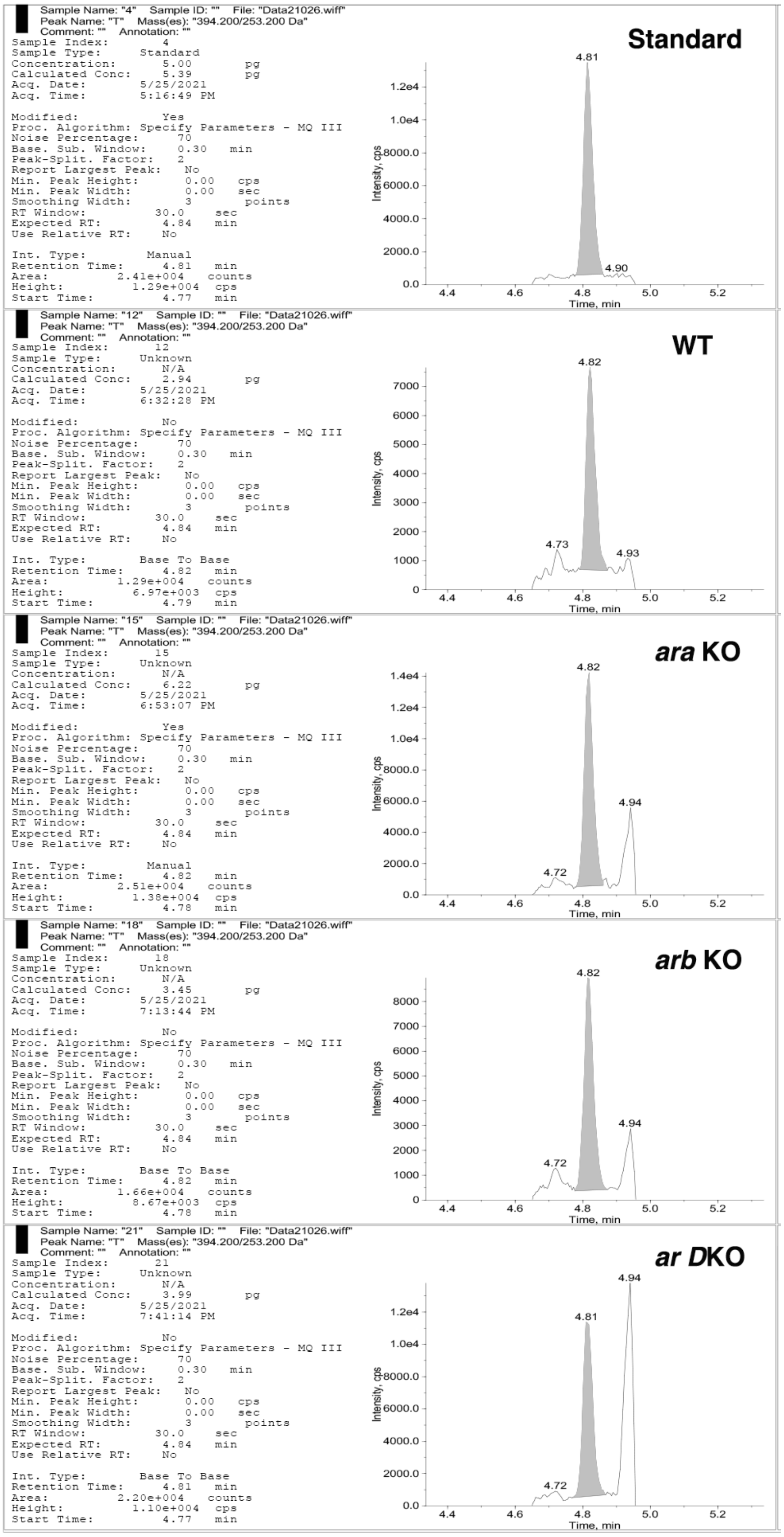

d Chromatograms of T in a standard and brain extracts (Internal standard). T-13C3 was used an internal standard to precisely scale and measure analyte concentration in LC-MS/MS sample analysis.

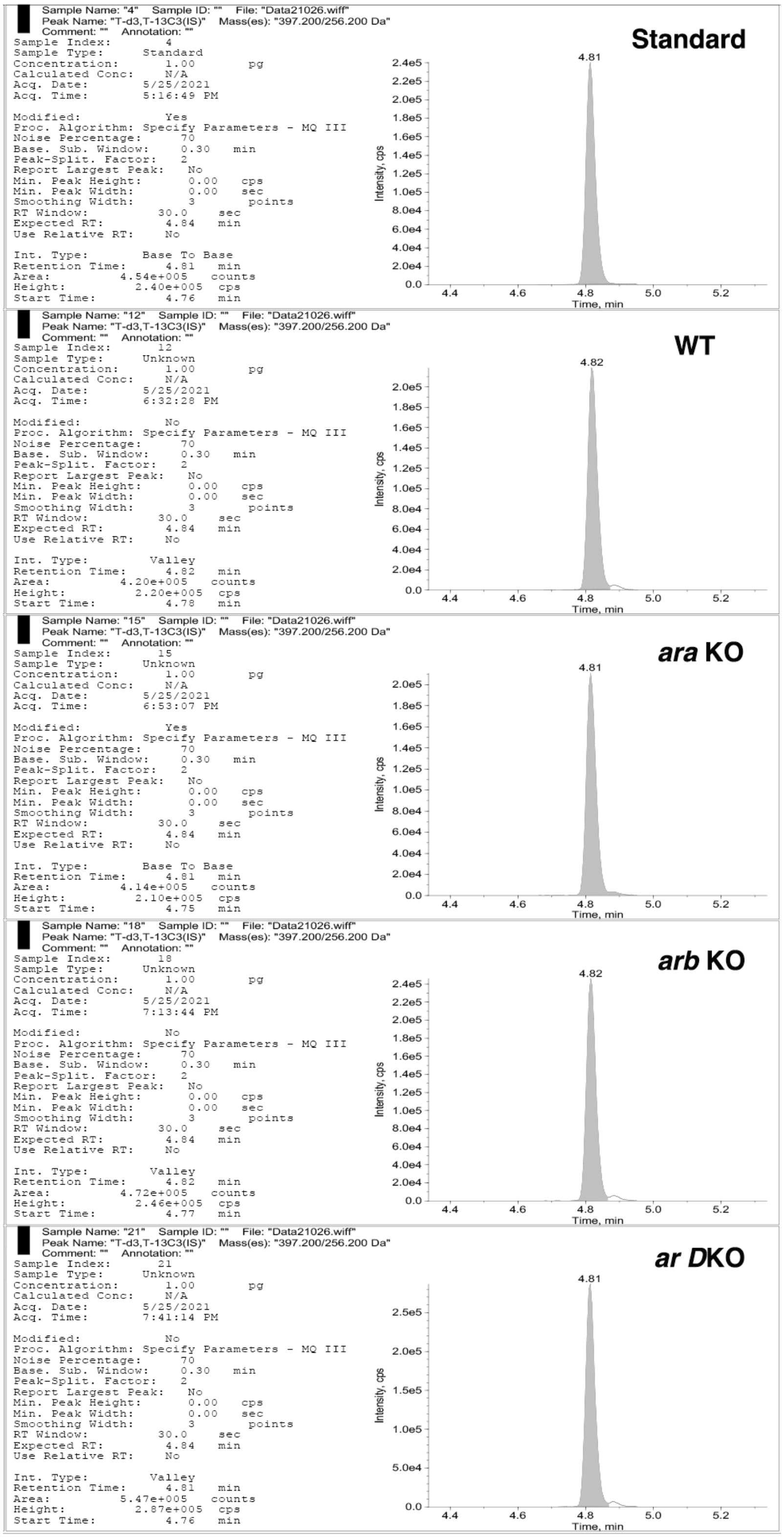

Supplementary Table 1.

Primers used in this study are listed.

name	Target	direction	purpose	sequence (5' to 3')
ARA-6F	*ara*	forward	Screening of mutant from TILLING Library, genotyping of knockouts (PCR on genomic DNA)	GTGTTTTCCATAACCGTGTG
ARA-6R	*ara*	reverse	Screening of mutant from TILLING Library, genotyping of knockouts (PCR on genomic DNA)	ACGGAAAACTTCCAAGACT
ARB-4F	*arb*	forward	Screening of mutant from TILLING Library, genotyping of knockouts (PCR on genomic DNA)	AACTATATGGAGTATATCAACAATCTTT
ARB-4R	*arb*	reverse	Screening of mutant from TILLING Library, genotyping of knockouts (PCR on genomic DNA)	CCAGTGTTTTTCATGCATCTdmy
DMY-5UF	*dmy*	forward	genotyping of mutants (PCR on genomic DNA)	CCGGGTGCCCAAGTGCTCCCGCTG
DMY-6SR	*dmy*	reverse	genotyping of mutants (PCR on genomic DNA)	GATCGTCCCTCCACAGAGAAGAGA
ARA-SM mut1	*ara*	forward	mutagenesis	CAGCTGTAGTGGATGGGGGTGATGGT
ARA-AM mut1	*ara*	reverse	mutagenesis	CATCCACTACAGCTGAATCACTGACA
ARB-SM mut1	*arb*	forward	mutagenesis	CAACATTTAGGAGTCCATTGAGCCAGA
ARB-AM mut1	*arb*	reverse	mutagenesis	GGACTCCTAAATGTTGAGGAAGACCA
ARA-LBD-F3	*ara*	forward	Confirmation of mutagenesis by sequencing	TGCTTATCCCTCCTGAGCATCC
ARB-DBD-F1	*arb*	forward	Confirmation of mutagenesis by sequencing	TGTCTGATCTGTTCAGATGAGGC
T7-sgRNA-OlARa-intron8-1	*ara*	forward	Amplification of DNA fragment for sgRNAs synthesis	TAATACGACTCACTATAggGAGGCTCTTTCAGCCGTGGTTTTAGAGCTAGAAATAGC
T7-sgRNA-OlARb-intron7-1	*arb*	forward	Amplification of DNA fragment for sgRNAs synthesis	TAATACGACTCACTATAggTGCAGTTGCTCAGGGGGAGTTTTAGAGCTAGAAATAGC
sgRNA-RV	*pDR274*	reverse	Amplification of DNA fragment for sgRNAs synthesis	AAAAGCACCGACTCGGTGCC
ara-KI-5FW	*ara*	forward	Confirmation of insertion of donor vector (genotyping of Ar-FLAG-mClover3 KI medaka strains)	TGCAGATCAACTGCTTTTCG
arb-KI-5FW	*arb*	forward	Confirmation of insertion of donor vector (genotyping of	TGTTGAGGGCCTGAAAAGTC

			Ar-FLAG-mClover3 KI medaka strains）	
GFP-127RV	*gfp*	reverse	Confirmation of insertion of donor vector（genotyping of Ar-FLAG-mClover3 KI medaka strains）	GCAGATGAACTTCAGGGTCAG
lef1-qF1	*lef1*	forward	real-time PCR	CGAGTGCACGCTGAAGGA
lef1-qR1	*lef1*	reverse	real-time PCR	CCTCCGTCCCAGGATCTGA
rpl7-qF1	*rpl-7*	forward	real-time PCR	CGCCAGATCTTCAACGGTGTAT
rpl7-qR1	*rpl-7*	reverse	real-time PCR	AGGCTCAGCAATCCTCAGCAT
gsdf-S1	*gsdf*	forward	RT-PCR for probe synthesis	TCCACCATGTCTTTGGCAC
gsdf-R1	*gsdf*	reverse	RT-PCR for probe synthesis	TGACCAACCCCTGCCTAC
vasa-S1	*vasa*	forward	RT-PCR for probe synthesis	ACGGCCCAAAGTGACCTAC
vasa-R1	*vasa*	reverse	RT-PCR for probe synthesis	GGGTCGTAGAAGGACACGG
P450c17-S1	*P450c17*	forward	RT-PCR for probe synthesis	CTCTGTGCTCCACCCTGT
P450c17-R1	*P450c17*	reverse	RT-PCR for probe synthesis	GGTCTGGGTGTGGCTTTC
ara-F	*ara*	forward	Screening and genotyping of Talen mutation allele (PCR on genomic DNA)	TGTTTGTTCTCTGCGCACTC
ara-R	*ara*	reverse	Screening and genotyping of Talen mutation allele (PCR on genomic DNA)	TGGGATCCTTGCAGATGAAT
arb-F	*arb*	forward	Screening and genotyping of Talen mutation allele (PCR on genomic DNA)	GCAGCAGAAACTGCTCACAG
arb-R	*arb*	reverse	Screening and genotyping of Talen mutation allele (PCR on genomic DNA)	GACACCTGTACTCGGCCACT

Supplementary Table. 2
Number of the behavioural tests utilized for the analysis

	Total number of behavioural tests	Successfully mated cases / Total tests (Fig 1a)	Cases with courtship display / total tests (Fig 5a)	Tests analyzed for Fig 5b-e****.
WT	60<br>(n = 11)	55/60	59/59*	55<br>(n = 11)
ara KO	76<br>(n = 14)	38/76	71/76	38<br>(n = 13***)
WT	42<br>(n = 6)	42/42	39/40**	42<br>(n = 6)
arb KO	41<br>(n = 6)	32/41	38/40*	32<br>(n = 6)
ar DKO	43<br>(n = 10)	0/43	0/43	0<br>(n = 10)

***We did not include one test in which a male successfully mated without courtship display but the recording time was shorter than 30 min.**
**** We did not include two tests in which males successfully mated without courtship display but the recording time was shorter than 30 min.**
*****We did not include one male who did not successfully mate with a female within 30 min.**
****** We used only the successfully mated cases for the analyses shown in Fig 5b-e.**

Supplementary Table 3.

Statistical data for each experiment are summarized.

Signif. codes: 0 '***' 0.001 '**' 0.01 '*' 0.05 '.' 0.1 ' ' 1

Fig. 1a

Fisher's exact test with Bonferroni's correction

Group1	Group2	Corrected *P* value	
ara WT	ara KO	9.96E-07	***
ara WT	arb WT	0.7578	
ara WT	arb KO	0.772	
ara WT	DKO	8.69E-23	***
ara KO	arb WT	8.59E-09	***
ara KO	arb KO	0.03281	*
ara KO	DKO	4.14E-09	***
arb WT	arb KO	0.01066	*
arb WT	DKO	3.01E-24	***
arb KO	DKO	2.24E-14	***

Fig. 1b

A binomial genelized mixed model followed by Dunnett test

		Estimate	Std. Error	t value	Pr(>|t|)	
araKO	WT	-2.97	0.5503	-5.397	1.35E-07	***
arbKO	WT	-4.1406	0.1659	-24.964	< 1e-10	***

Fig. 1c

ANOVA	Df	Sum Sq	Mean Sq	F value	Pr(>F)	
genotype	3	3.09	1.0298	13.16	0.000137	***
Residuals	16	1.252	0.0782			

Dunnett test		Estimate	Std. Error	t value	Pr(>|t|)	
araKO	WT	0.7785	0.1694	4.597	<0.001	***
arbKO	WT	-0.1208	0.1694	-0.713	0.827	
arDKO	WT	-0.2493	0.1805	-1.381	0.41	

Fig. 1f

ANOVA	Df	Sum Sq	Mean Sq	F value	Pr(>F)	
genotype	3	93.46	31.154	5.731	0.00735	**
Residuals	16	86.97	5.436			

Dunnett test		Estimate	Std. Error	t value	Pr(>|t|)	
araKO	WT	1.7474	1.4118	1.238	4.94E-01	
arbKO	WT	-0.4213	1.4118	-0.298	0.9829	
arDKO	WT	5.4161	1.5049	3.599	0.0067	**

Fig. 1g

ANOVA	Df	Sum Sq	Mean Sq	F value	Pr(>F)
genotype	3	300.6	100.2	0.799	0.513
Residuals	16	2007.3	125.5		

Dunnett test		Estimate	Std. Error	t value	Pr(>|t|)
araKO	WT	3.386	6.782	0.499	0.929
arbKO	WT	-4.888	6.782	-0.721	0.823
arDKO	WT	-6.777	7.23	-0.937	0.689

Fig. 1h

ANOVA	Df	Sum Sq	Mean Sq	F value	Pr(>F)
genotype	3	256.7	85.57	1.365	0.289
Residuals	16	1002.9	62.68		

Dunnett test		Estimate	Std. Error	t value	Pr(>|t|)
araKO	WT	4.175	4.794	0.871	0.732
arbKO	WT	-3.849	4.794	-0.803	0.775
arDKO	WT	5.569	5.11	1.09	0.589

Fig. 1i

A binomial genelized mixed model followed by Dunnett test

		Estimate	Std. Error	t value	Pr(>|t|)
araKO	WT	-12.381	6.328	-1.956	0.173
arbKO	WT	-6.443	6.328	-1.018	0.626
arDKO	WT	-11.25	6.328	-1.778	0.229

Signif. codes: 0 '***' 0.001 '**' 0.01 '*' 0.05 '.' 0.1 ' ' 1

Fig. 2e

ANOVA	Df	Sum Sq	Mean Sq	F value	Pr(>F)	
genotype	3	36513	12171	447.7	<2e-16	***
Residuals	32	870	27			
Dunnett test		Estimate	Std. Error	t value	Pr(>|t|)	
araKO	WT	2.667	2.458	1.085	0.572	
arbKO	WT	-62.333	2.458	-25.36	<1e-04	***
arDKO	WT	-62.333	2.458	-25.36	<1e-04	***

Fig. 2f (3rd fin ray)

ANOVA	Df	Sum Sq	Mean Sq	F value	Pr(>F)	
genotype	3	2885.8	961.9	52.46	2.83E-16	***
sex	1	681.6	681.6	37.17	1.06E-07	***
genotype:sex	3	1218.1	406	22.14	1.39E-09	***
Residual	56	1026.9	18.3			
Tukey test						
Group1	Group2	Estimate	Std. Error	t value	Pr(>|t|)	
araKO male	WT male	-4.0402	2.1411	-1.887	0.56506	
arbKO male	WT male	-24.0451	2.1411	-11.23	<0.001	***
arDKO male	WT male	-23.3389	2.1411	-10.9	<0.001	***
WT female	WT male	-18.6946	2.1411	-8.731	<0.001	***
araKO female	WT male	-14.9403	2.1411	-6.978	<0.001	***
arbKO female	WT male	-22.8223	2.1411	-10.659	<0.001	***
arDKO female	WT male	-21.0743	2.1411	-9.843	<0.001	***
arbKO male	araKO male	-20.0049	2.1411	-9.343	<0.001	***
arDKO male	araKO male	-19.2987	2.1411	-9.013	<0.001	***
WT female	araKO male	-14.6544	2.1411	-6.844	<0.001	***
araKO female	araKO male	-10.9001	2.1411	-5.091	<0.001	***
arbKO female	araKO male	-18.7821	2.1411	-8.772	<0.001	***
arDKO female	araKO male	-17.0341	2.1411	-7.956	<0.001	***
arDKO male	arbKO male	0.7062	2.1411	0.33	0.99998	
WT female	arbKO male	5.3505	2.1411	2.499	0.21735	
araKO female	arbKO male	9.1048	2.1411	4.252	0.00203	**
arbKO female	arbKO male	1.2227	2.1411	0.571	0.99909	
arDKO female	arbKO male	2.9708	2.1411	1.387	0.85909	
WT female	arDKO male	4.6443	2.1411	2.169	0.38565	
araKO female	arDKO male	8.3986	2.1411	3.923	0.00555	**
arbKO female	arDKO male	0.5165	2.1411	0.241	1	
arDKO female	arDKO male	2.2646	2.1411	1.058	0.96279	
araKO female	WT female	3.7543	2.1411	1.753	0.65282	
arbKO female	WT female	-4.1277	2.1411	-1.928	0.53831	
arDKO female	WT female	-2.3797	2.1411	-1.111	0.95163	
arbKO female	araKO female	-7.8821	2.1411	-3.681	0.01148	*
arDKO female	araKO female	-6.134	2.1411	-2.865	0.09959	.
arDKO female	arbKO female	1.748	2.1411	0.816	0.99147	

> cld(fig2f.A3rd.Tukey, level = 0.05, decreasing = TRUE)

WT.male	araKO.male	arbKO.male	arDKO.male	WT.female	araKO.female
"a"	"a"	"c"	"c"	"bc"	"b"

arbKO.female	arDKO.female
"c"	"bc"

Fig. 2f (Posterior 2nd fin ray)

ANOVA	Df	Sum Sq	Mean Sq	F value	Pr(>F)	
genotype	3	1508	502.7	26.38	9.06E-11	***
sex	1	1730.6	1730.6	90.83	2.53E-13	***
genotype:sex	3	696.1	232	12.18	3.05E-06	***
Residual	56	1066.9	19.1			

Tukey test

Group1	Group2	Estimate	Std. Error	t value	Pr(>|t|)	
araKO male	WT male	0.3616	2.1825	0.166	1	
arbKO male	WT male	-13.5514	2.1825	-6.209	<0.001	***
arDKO male	WT male	-16.6536	2.1825	-7.631	<0.001	***
WT female	WT male	-17.3085	2.1825	-7.931	<0.001	***
araKO female	WT male	-14.5173	2.1825	-6.652	<0.001	***
arbKO female	WT male	-22.8076	2.1825	-10.45	<0.001	***
arDKO female	WT male	-16.8104	2.1825	-7.702	<0.001	***
arbKO male	araKO male	-13.913	2.1825	-6.375	<0.001	***
arDKO male	araKO male	-17.0151	2.1825	-7.796	<0.001	***
WT female	araKO male	-17.6701	2.1825	-8.096	<0.001	***
araKO female	araKO male	-14.8789	2.1825	-6.817	<0.001	***
arbKO female	araKO male	-23.1691	2.1825	-10.616	<0.001	***
arDKO female	araKO male	-17.172	2.1825	-7.868	<0.001	***
arDKO male	arbKO male	-3.1022	2.1825	-1.421	0.84364	
WT female	arbKO male	-3.7571	2.1825	-1.722	0.67348	
araKO female	arbKO male	-0.9659	2.1825	-0.443	0.99983	
arbKO female	arbKO male	-9.2562	2.1825	-4.241	0.00202	**
arDKO female	arbKO male	-3.259	2.1825	-1.493	0.80765	
WT female	arDKO male	-0.6549	2.1825	-0.3	0.99999	
araKO female	arDKO male	2.1363	2.1825	0.979	0.97565	
arbKO female	arDKO male	-6.154	2.1825	-2.82	0.11047	
arDKO female	arDKO male	-0.1568	2.1825	-0.072	1	
araKO female	WT female	2.7912	2.1825	1.279	0.90287	
arbKO female	WT female	-5.4991	2.1825	-2.52	0.2085	
arDKO female	WT female	0.4981	2.1825	0.228	1	
arbKO female	araKO female	-8.2903	2.1825	-3.799	0.00809	**
arDKO female	araKO female	-2.2931	2.1825	-1.051	0.96409	
arDKO female	arbKO female	5.9972	2.1825	2.748	0.13045	

> cld(fig2f.P2nd.Tukey, level = 0.05, decreasing = TRUE)

WT.male	araKO.male	arbKO.male	arDKO.male	WT.female	araKO.female
a	a	b	bc	bc	b

arbKO.female	arDKO.female
c	bc

Signif. codes: 0 '***' 0.001 '**' 0.01 '*' 0.05 '.' 0.1 ' ' 1

Fig. 4d

ANOVA	Df	Sum Sq	Mean Sq	F value	Pr(>F)	
genotype	2	0.01778	0.00889	3.796	0.041	*
Residuals	19	0.0445	0.002342			

Dunnett test		Estimate	Std. Error	t value	Pr(>|t|)	
araKO	WT	-0.07172	0.02614	-2.744	0.0241	*
arbKO	WT	-0.02544	0.0242	-1.051	0.4872	

Tukey test

Group1	Group2	Estimate	Std. Error	t value	Pr(>|t|)	
araKO	WT	-0.07172	0.02614	-2.744	0.0327	*
arbKO	WT	-0.02544	0.0242	-1.051	0.5544	
arbKO	araKO	0.04628	0.02614	1.771	0.2058	

Signif. codes: 0 '***' 0.001 '**' 0.01 '*' 0.05 '.' 0.1 ' ' 1

Fig. 5a

Fisher's exact test with Bonferroni's correction

Group1	Group2	Corrected *P* value
ara WT	ara KO	0.351
ara WT	arb WT	1
ara WT	arb KO	1
ara WT	DKO	6.64E-22 ***
ara KO	arb WT	1
ara KO	arb KO	1
ara KO	DKO	2.52E-18 ***
arb WT	arb KO	1
arb WT	DKO	6.54E-16 ***
arb KO	DKO	6.54E-16 ***

Fig. 5b

Likelihood ratio test

null model: courtship ~ 1 + (1 | id)
full model: courtship ~ genotype + (1 | id)

>ara	npar	AIC	BIC	logLik	deviance	Chisq	Df	Pr(>Chisq)
null model:	2	616.04	621.11	-306.02	612.04			
full model:	3	617.14	624.74	-305.57	611.14	0.9035	1	0.3419
>arb	npar	AIC	BIC	logLik	deviance	Chisq	Df	Pr(>Chisq)
null model:	2	479.31	483.92	-237.66	475.31			
full model:	3	476.75	483.66	-235.38	470.75	4.5623	1	0.03268 *

Fig. 5c

Likelihood ratio test

null model: mating_latency ~ 1 + (1 | id)
full model: mating_latency ~ genotype + (1 | id)

>ara	npar	AIC	BIC	logLik	deviance	Chisq	Df	Pr(>Chisq)
null model:	3	1344.9	1352.5	-669.44	1338.9			
full model:	4	1342.3	1352.4	-667.13	1334.3	4.6182	1	0.03163 *
>arb	npar	AIC	BIC	logLik	deviance	Chisq	Df	Pr(>Chisq)
null model:	3	996.72	1003.63	-495.36	990.72			
full model:	4	988.45	997.67	-490.22	980.45	10.268	1	0.001353 **

Fig. 5d

Likelihood ratio test

null model: wrap_rejection ~ 1 + (1 | id)
full model: wrap_rejection ~ genotype + (1 | id)

>ara	npar	AIC	BIC	logLik	deviance	Chisq	Df	Pr(>Chisq)
null model:	2	417.67	422.74	-206.84	413.67			
full model:	3	394.13	401.72	-194.06	388.13	25.546	1	4.32E-07 ***
>arb	npar	AIC	BIC	logLik	deviance	Chisq	Df	Pr(>Chisq)
null model:	2	312.81	317.41	-154.4	308.81			
full model:	3	305.91	312.82	-149.95	299.91	8.8961	1	0.002858 **

Fig. 5e

Likelihood ratio test

null model: wrap_duration ~ 1 + (1 | id)
full model: wrap_duration ~ genotype + (1 | id)

>ara	npar	AIC	BIC	logLik	deviance	Chisq	Df	Pr(>Chisq)
null model:	3	668.67	676.27	-331.34	662.67			
full model:	4	660.8	670.93	-326.4	652.8	9.875	1	0.001675 **
>arb	npar	AIC	BIC	logLik	deviance	Chisq	Df	Pr(>Chisq)
null model:	3	478.1	485.01	-236.05	472.1			
full model:	4	476.04	485.26	-234.02	468.04	4.0553	1	0.04403 *

Signif. codes: 0 '***' 0.001 '**' 0.01 '*' 0.05 '.' 0.1 ' ' 1

Table 1. LC/MS analysis of androgen and estrogen in testes

T

ANOVA	Df	Sum Sq	Mean Sq	F value	Pr(>F)	
genotype	3	76.43	25.48	1.352	0.325	
Residuals	8	150.75	18.84			
Dunnett test		Estimate	Std. Error	t value	Pr(>|t|)	
araKO	WT	3.9967	3.5444	1.128	0.565	
arbKO	WT	0.9833	3.5444	0.277	0.985	
arDKO	WT	-3.0733	3.5444	-0.867	0.727	

11KT

ANOVA	Df	Sum Sq	Mean Sq	F value	Pr(>F)	
genotype	3	7983	2661	26.58	0.000164	***
Residuals	8	801	100.1			
Dunnett test		Estimate	Std. Error	t value	Pr(>|t|)	
araKO	WT	15.317	8.17	1.875	0.218	
arbKO	WT	6.267	8.17	0.767	0.788	
arDKO	WT	65.417	8.17	8.007	<0.001	***

E2

ANOVA	Df	Sum Sq	Mean Sq	F value	Pr(>F)	
genotype	3	1.382	0.4607	2.007	0.192	
Residuals	8	1.836	0.2295			
Dunnett test		Estimate	Std. Error	t value	Pr(>|t|)	
araKO	WT	-0.72	0.3912	-1.841	0.229	
arbKO	WT	-0.9067	0.3912	-2.318	0.115	
arDKO	WT	-0.5967	0.3912	-1.525	0.351	

Signif. codes: 0 '***' 0.001 '**' 0.01 '*' 0.05 '.' 0.1 ' ' 1

Table 2. LC/MS analysis of androgen and estrogen in brains

T

ANOVA	Df	Sum Sq	Mean Sq	F value	Pr(>F)	
genotype	3	262574	87525	24.42	0.000222	**
Residuals	8	28677	3585			
Dunnett test		Estimate	Std. Error	t value	Pr(>|t|)	
araKO	WT	167.03	48.89	3.417	0.0225	*
arbKO	WT	-57.2	48.89	-1.17	0.5393	
arDKO	WT	320.33	48.89	6.553	<0.001	**

11KT

ANOVA	Df	Sum Sq	Mean Sq	F value	Pr(>F)	
genotype	3	144.55	48.18	340.9	8.90E-09	**
Residuals	8	1.13	0.14			
Dunnett test		Estimate	Std. Error	t value	Pr(>|t|)	
araKO	WT	1.373	0.307	4.474	0.00524	**
arbKO	WT	0.03	0.307	0.098	0.99928	
arDKO	WT	8.38	0.307	27.3	<0.001	**

E2

ANOVA	Df	Sum Sq	Mean Sq	F value	Pr(>F)	
genotype	3	22572	7524	4.724	0.0352	*
Residuals	8	12743	1593			
Dunnett test		Estimate	Std. Error	t value	Pr(>|t|)	
araKO	WT	-20.17	32.59	-0.619	0.8693	
arbKO	WT	-94.7	32.59	-2.906	0.0479	*
arDKO	WT	-96.57	32.59	-2.963	0.0442	*

E1

ANOVA	Df	Sum Sq	Mean Sq	F value	Pr(>F)	
genotype	3	52344	17448	6.351	0.0164	*
Residuals	8	21978	2747			
Dunnett test		Estimate	Std. Error	t value	Pr(>|t|)	
araKO	WT	45.77	42.8	1.069	0.6006	
arbKO	WT	-127.57	42.8	-2.981	0.0431	*
arDKO	WT	-69.07	42.8	-1.614	0.3124	

Supplementary Fig. 2d

Mann-Whitney U test

	W	*P* value
ara (WT vs S507X)	16	0.02857
arb (WT vs L503X)	16	0.02857

Signif. codes: 0 '***' 0.001 '**' 0.01 '*' 0.05 '.' 0.1 ' ' 1

Supplementary Fig. 3a

ANOVA	Df		Sum Sq	Mean Sq	F value	Pr(>F)
genotype		3	218.2	72.73	1.317	0.314
Residuals		12	662.7	55.23		
Dunnett test			Estimate	Std. Error	t value	Pr(>|t|)
araKO	WT		7.25	5.255	1.38	0.407
arbKO	WT		-0.5	5.255	-0.095	0.999
arDKO	WT		7	5.255	1.332	0.433

Supplementary Fig. 3b

ANOVA	Df		Sum Sq	Mean Sq	F value	Pr(>F)
genotype		3	1119	372.9	1.072	0.398
Residuals		12	4175	347.9		
Dunnett test			Estimate	Std. Error	t value	Pr(>|t|)
araKO	WT		-17.5	13.19	-1.327	0.436
arbKO	WT		-20	13.19	-1.516	0.338
arDKO	WT		-20	13.19	-1.516	0.338

Supplementary Fig. 3c

ANOVA	Df		Sum Sq	Mean Sq	F value	Pr(>F)
genotype		3	3228	1076	0.65	0.598
Residuals		12	19883	1657		
Dunnett test			Estimate	Std. Error	t value	Pr(>|t|)
araKO	WT		-13.25	28.78	-0.46	0.938
arbKO	WT		-34.75	28.78	-1.207	0.506
arDKO	WT		-32	28.78	-1.112	0.566

Signif. codes: 0 '***' 0.001 '**' 0.01 '*' 0.05 '.' 0.1 ' ' 1

Supplementary Fig. 6

ANOVA	Df		Sum Sq	Mean Sq	F value	Pr(>F)	
genotype		3	385.5	128.49	6.355	0.0164	*
Residuals		8	161.8	20.22			
Dunnett test			Estimate	Std. Error	t value	Pr(>|t|)	
araKO	WT		-4.013	3.671	-1.093	0.58592	
arbKO	WT		-9.84	3.671	-2.68	0.06707	.
arDKO	WT		-14.915	3.671	-4.062	0.00931	**

Signif. codes: 0 '***' 0.001 '**' 0.01 '*' 0.05 '.' 0.1 ' ' 1

Supplementary Fig. 7

χ^2 test	Df	χ2	P value	
ara WT vs KC	1	23.458	1.28E-06	***
arb WT vs KC	1	12.224	0.0004718	***

Supplementary References

1. Miyagawa S*, et al.* Dosage-dependent hedgehog signals integrated with Wnt/beta-catenin signaling regulate external genitalia formation as an appendicular program. *Development* **136**, 3969-3978 (2009).
2. Ansai S*, et al.* Efficient targeted mutagenesis in medaka using custom-designed transcription activator-like effector nucleases. *Genetics* **193**, 739-749 (2013).
3. Matsuda M*, et al.* DMY gene induces male development in genetically female (XX) medaka fish. *Proc Natl Acad Sci U S A* **104**, 3865-3870 (2007).
4. Ogino Y*, et al.* Neofunctionalization of androgen receptor by gain-of-function mutations in teleost fish lineage. *Mol Biol Evol* **33**, 228-244 (2016)
5. Li Y, Liu Y, Yang H, Zhang T, Naruse K, Tu Q. Dynamic transcriptional and chromatin accessibility landscape of medaka embryogenesis. *Genome Res* **30**, 924-937 (2020).
6. Schwartz S*, et al.* MultiPipMaker and supporting tools: Alignments and analysis of multiple genomic DNA sequences. *Nucleic Acids Res* **31**, 3518-3524 (2003).
7. Kent WJ*, et al.* The human genome browser at UCSC. *Genome Res* **12**, 996-1006 (2002).
8. Martin FJ*, et al.* Ensembl 2023. *Nucleic Acids Res* (2022).
