# Supplementary material for: Evolutionary differentiation of androgen receptor is responsible for sexual characteristic development in a teleost fish
Source: Nat Commun. 2023 Mar 14;14:1428. doi: 10.1038/s41467-023-37026-6 (PMC10014959; doi:10.1038/s41467-023-37026-6)
Supplement: Supplementary file 2 — Description of Additional Supplementary Files [file 41467_2023_37026_MOESM2_ESM.pdf]

Description of Additional Supplementary Files

File Name: Supplementary Movie 1
Description: Aggressive male-male competition
Two WT males were placed in the same tank with a WT female, and recorded from the top of the tank using a high-speed camera system (HAS-L2, DITECT) at 300 frames per second with a resolution of 800×600 pixels. This movie file was made by a slow-motion replay at 30 frames per second.

File Name: Supplementary Movie 2
Description: The mating behavioural test (an *ara* WT male and a WT female, short version of a representative case, file size < 50MB).
Mating behaviour was recorded from the side of the tank using a digital video camera HDR-PJ800 (Sony, Tokyo, Japan). This movie file is from video ID: MAH00178-2.

File Name: Supplementary Movie 3
Description: The mating behavioural test (an *ara* KO male and a WT female, short version of a representative case, file size < 50MB).
Mating behaviour was recorded from the side of the tank using a digital video camera HDR-PJ800 (Sony, Tokyo, Japan). This movie file is from video ID: MAH00295.

File Name: Supplementary Movie 4
The mating behavioural test (an *arb* WT male and a WT female, short version of a representative case, file size < 50MB).
Mating behaviour was recorded from the side of the tank using a digital video camera HDR-PJ800 (Sony, Tokyo, Japan). This movie file is from video ID: MAH00157.

File Name: Supplementary Movie 5
Description: The mating behavioural test (an *arb* KO male and a WT female, short version of a representative case, file size < 50MB).
Mating behaviour was recorded from the side of the tank using a digital video camera HDR-PJ800 (Sony, Tokyo, Japan). This movie file is from video ID: MAH00158.

File Name: Supplementary Movie 6
Description: The mating behavioural test (an *ar* DKO male and a WT female, short version of a representative case, file size < 50MB).
Mating behaviour was recorded from the side of the tank using a digital video camera

HDR-PJ800 (Sony, Tokyo, Japan). This movie file is from video ID: MAH00291-2.
The list of video ID was included in the source data file.
The remaining video data are available from the authors upon request.
