# Supplementary material for: Evolutionary differentiation of androgen receptor is responsible for sexual characteristic development in a teleost fish
Source: Nat Commun. 2023 Mar 14;14:1428. doi: 10.1038/s41467-023-37026-6 (PMC10014959; doi:10.1038/s41467-023-37026-6)
Supplement: Supplementary file 10 — Reporting Summary [file 41467_2023_37026_MOESM10_ESM.pdf]

nature portfolio

Corresponding author(s): Yukiko Ogino

Last updated by author(s): Feb 11, 2023

Reporting Summary

Nature Portfolio wishes to improve the reproducibility of the work that we publish. This form provides structure for consistency and transparency in reporting. For further information on Nature Portfolio policies, see our Editorial Policies and the Editorial Policy Checklist.

Statistics

For all statistical analyses, confirm that the following items are present in the figure legend, table legend, main text, or Methods section.

n/a	Confirmed	
☐	☒	The exact sample size (n) for each experimental group/condition, given as a discrete number and unit of measurement
☐	☒	A statement on whether measurements were taken from distinct samples or whether the same sample was measured repeatedly
☐	☒	The statistical test(s) used AND whether they are one- or two-sided<br>*Only common tests should be described solely by name; describe more complex techniques in the Methods section.*
☐	☒	A description of all covariates tested
☐	☒	A description of any assumptions or corrections, such as tests of normality and adjustment for multiple comparisons
☐	☒	A full description of the statistical parameters including central tendency (e.g. means) or other basic estimates (e.g. regression coefficient) AND variation (e.g. standard deviation) or associated estimates of uncertainty (e.g. confidence intervals)
☐	☒	For null hypothesis testing, the test statistic (e.g. F, t, r) with confidence intervals, effect sizes, degrees of freedom and P value noted<br>*Give P values as exact values whenever suitable.*
☒	☐	For Bayesian analysis, information on the choice of priors and Markov chain Monte Carlo settings
☒	☐	For hierarchical and complex designs, identification of the appropriate level for tests and full reporting of outcomes
☐	☒	Estimates of effect sizes (e.g. Cohen's d, Pearson's r), indicating how they were calculated

Our web collection on statistics for biologists contains articles on many of the points above.

Software and code

Policy information about availability of computer code

Data collection	Data for sperm motility, average speed, and sperm concentration were obtained using CASA (SMAS3(ver.3.1.11.357), DITECT, Tokyo, Japan). Gross morphology, tissue and immunohistochemical data were obtained by using a digital charge-coupled device camera DP-73 (Olympus, Tokyo, Japan). The lengths of the fin and tooth of each fish were calculated using Adobe Photoshop CC 2019 (ver. 20.0.2) using a picture taken with DP-73. The micro CT-scanned image was taken by Phoenix nanotom m (Baker Hughes, Houston, TX) at the JMC Corporation (Yokohama, Japan). The videos for mating behaviour and mate-choice test were recorded for 30 min using a digital video camera HDR-PJ800, (Sony, Tokyo, Japan). The movie of aggressive male-male competition was taken using a high-speed camera system (HAS-L2, DITECT).
Data analysis	For the screening of the Ar mutants, we used the LightScanner 96 software v2.0. The statistical analysis for the frequency of reproduction and the frequency of mating that fish exhibited courtship display was conducted two-sided fisher's exact test with a Bonferroni correction using R version 4.2.0. For the other behavioral data, the statistical analysis was done using generalized liner mixed models (GLMMs) and linear mixed models (LMMs) in R version 4.2.0 with the package lme4 version 1.1.30. For the mate choice test, the statistics was analysed using the chi-squared (χ2) test of independence in R version 4.2.0 with the package lme4 version 1.1.30. For RNA-seq, the adapter and quality trimming was done by using Trim Galore 0.6.4_dev with Cutadapt 1.18, and then the transcripts were quantified using R. package tximport and salmon v1.3.0 by mapping the trimmed reads to the transcriptome sequences of Oryzias latipes (Hd-rR; ASM223467v1) with the Ensembl gene annotation (Release 105; https://www.ensembl.org/). The statistical analysis for the RNA-seq data was performed using the R package edgeR v3.34.1 and the gene ontology (GO) enrichment analysis was conducted using ShinyGO version 0.75 (http://bioinformatics.sdstate.edu/go/) using the Ensembl gene IDs of the differentially expressed genes as input. For qPCR primer design, we used Applied Biosystems Primer Express 2.0.0 (Thermo Fisher Scientific). The statistical analysis of GSI, sperm motility, number of papillary processes, LC/MS, female fecundity, and qRT-PCR data, was done by the one-way ANOVA followed by two-sided Dunnett's multiple comparison test in the 'glht' function of the R package multcomp version 1.4-20. The statistical analysis of fertilization rate for natural mating and artificial insemination was done by the two-sided Dunnett's multiple comparison in binomial GLMMs in R version 4.2.0 with the packages lme4 version 1.1-31 and multicomp version

1.4-20. For the statistical analysis of fin length, we used the two-way ANOVA followed by two-sided Tukey's multiple comparison test in the 'glht' function of the R package multcomp version 1.4-20. For the analysis of tooth length, we used the one-way ANOVA followed by two-sided Tukey's multiple comparison test in the 'glht' function of the R package multcomp version 1.4-20. For the analysis of reporter gene assay, we used two-sided Mann-Whitney U test by R version 4.2.0. We created all box plots using R version 3.6.2 and ggplot2 package. For the comparison of cis-regulatory sequences of ar genes, we used MultiPipMaker web version (http://pipmaker.bx.psu.edu/pipmaker/).

For manuscripts utilizing custom algorithms or software that are central to the research but not yet described in published literature, software must be made available to editors and reviewers. We strongly encourage code deposition in a community repository (e.g. GitHub). See the Nature Portfolio guidelines for submitting code & software for further information.

Data

Policy information about availability of data

All manuscripts must include a data availability statement. This statement should provide the following information, where applicable:

- Accession codes, unique identifiers, or web links for publicly available datasets
- A description of any restrictions on data availability
- For clinical datasets or third party data, please ensure that the statement adheres to our policy

RNA-seq data for the whole brain with a pituitary gland are available from DDBJ (Accession No. DRA013672). The transcriptome sequences of Oryzias latipes (Hd-rR; ASM223467v1)in Ensembl Release 100 (http://dec2021.archive.ensembl.org/) were used for the RNA-seq analysis. The cDNA sequences for medaka ars are available from Genbank (Accession No. AB252233 for ara; AB252679 for arb). The genome sequences for medaka ars are available from Ensembl (ID: ENSORLG00000008220 for ara; ENSORLG00000009520 for arb).

Human research participants

Policy information about studies involving human research participants and Sex and Gender in Research.

Reporting on sex and gender	We do not include any human data in our manuscript.
Population characteristics	See above.
Recruitment	See above.
Ethics oversight	See above.

Note that full information on the approval of the study protocol must also be provided in the manuscript.

Field-specific reporting

Please select the one below that is the best fit for your research. If you are not sure, read the appropriate sections before making your selection.

☒ Life sciences ☐ Behavioural & social sciences ☐ Ecological, evolutionary & environmental sciences

For a reference copy of the document with all sections, see nature.com/documents/nr-reporting-summary-flat.pdf

Life sciences study design

All studies must disclose on these points even when the disclosure is negative.

Sample size	No statistical method was used to predetermine sample size. We used lab-raised fish with expected small variability and were interested in large differences in gene expression and phenotypes between wild type males and Ar KO males, the most experiments except micro-CT images were performed on n≥3 to ensure reproducibility between replicates. The micro-CT-image was obtained from a single specimen in each genotype because of the large expense of performing. To ensure the reproducibility of the data of tooth morphology taken by micro-CT, we performed the bone staining of the teeth by using multiple fishes (n = 9 for each genotype).<br>For RNA-seq, we used three individuals for each sample as the recommended minimum number of replicate in previous publications (e.g. Lamarre et al. 2018 Front Plant Sci). For experiments to quantify the gene expression levels, sample sizes were determined based on the expected variability and the number of fish available according to other studies with similar methodology (e.g. Ogino et al. 2014 Endocrinology). For behavioral experiments, we used as many fish as available at least 6 males for each genotype. To dilute the biological variance of the female condition, we conducted at least three mating tests with different females for each male. For the calculation of fertilization rate in natural mating, we used ≥ 6 of males for each genotype. To reduce the biological variances of female condition, each male was mated at least twice with a different female. For the artificial insemination, we used 4 of males for each genotype. For the analysis of sperm mitility using CASA, we used ≥ 4 males for each genotype. For female fecundity, we used 4 females for each genotype. To dilute the biological variances of male condition, we collected the data for 10 days by mating with different males. The number of replications were sufficient to allow statistical testing.
Data exclusions	In the mating experiment of ara KO and arb KO males, the pairs that did not spawn eggs within the 30 min test were excluded from the data collection regarding the frequency of courtship display, mating latency, total number of wrapping rejection, and duration of wrapping with spawning, because such female may not have been ready for spawning. In other experiments, no data were excluded.

Replication	Unless otherwise stated, each experiment was conducted using multiple sample fishes as biological replicates. For RNA-seq, we used 3 fishes per sample with three biological replicates. For qPCR, we used 1 fish per sample with three biological replicates.<br>For all images of the knockout and knock-in fishes and their tissues, we used multiple fishes (n≥3 in each genotype) except micro-CT image. The micro-CT-image was obtained from a single specimen in each genotype. To confirm the data of tooth morphology taken by micro-CT, we performed the bone staining of the teeth by using multiple fishes (n = 9 for each genotype). For male behavioral experiments, we used at least 6 males for each genotype. To dilute the biological variance of the female condition, we conducted at least three mating tests with different females for each male. For female fecundity, we used 4 females for each genotype. To dilute the biological variance of male condition, we collected the data for 10 days by mating with different males. For the analysis of sperm motility using CASA, we used ≥ 4 fishes for each genotype and analyzed using the same parameter constant to ensure reproducibility. For reporter gene assay using COS-7 cells, data were collected by four independent experiments with means of three technical replicates. No experiments were unreplicated except the micro CT-image. No experiments were unable to be reproduced.
Randomization	In all experiments, each fish was randomly chosen from one of the families by confirming the genotype and age (i.e., family was randomized) and then allocated into the experimental group. In the behavioral experiments, each compartment was assigned randomly to the wild-type or the knockout fish.
Blinding	Investigators were not blinded to group allocation during the data collection, since the Investigators needed to be aware of genotypes of the medaka to perform all analysis.<br>Data analysis of behavioral experiments were conducted as blinded experiment.<br>Blinding is not relevant as the other analyses including RNA-seq and phenotype analyses of compared group were performed with the same pipelines and parameters and thus not subjected to observer bias.

Reporting for specific materials, systems and methods

We require information from authors about some types of materials, experimental systems and methods used in many studies. Here, indicate whether each material, system or method listed is relevant to your study. If you are not sure if a list item applies to your research, read the appropriate section before selecting a response.

Materials & experimental systems

n/a	Involved in the study	
☐	☒	Antibodies
☐	☒	Eukaryotic cell lines
☒	☐	Palaeontology and archaeology
☐	☒	Animals and other organisms
☒	☐	Clinical data
☒	☐	Dual use research of concern

Methods

n/a	Involved in the study	
☒	☐	ChIP-seq
☒	☐	Flow cytometry
☒	☐	MRI-based neuroimaging

Antibodies

Antibodies used	List of primary antibodies (format anti-protein (company, catlaog number, Clone number & Lot number)<br>1. mouse monoclonal anti-DDDDK-tag (FLAG) (MBL (Nagoya, Japan), M185-3S, Clone FLA-1, Lot 004),<br>2. rabbit monoclonal anti-GFP (Cell signaling (Danvers, MA, USA), 2956, Clone D5.1, Lot 2)<br><br>List of secondary antibodies ((format anti-protein (company, catalog number, Lot number)<br>1. goat anti-mouse IgG(H+L), F(ab')2 fragment, Alexa 555-conjugated (Cell signaling, 4409, Lot 18)<br>2. goat anti-rabbit IgG(H+L), F(ab')2 fragment, Alexa 488-conjugated (Cell signaling, 4412, Lot 4)
Validation	All the antibodies used in this study are commercially available, validated by the manufactures or by our laboratory:<br>1. mouse monoclonal anti-DDDDK-tag (FLAG) (https://ruo.mbl.co.jp/bio/e/dtl/A/?pcd=M185-3L)<br>The anti-DDDDK-tag (FLAG) mouse mAb reacts with the N-terminal, internal, and C-terminal DDDK-tgged (DYKDDDDK) proteins, whose availability for immunohistochemistry was validated by the manufacturer.<br>2. rabbit monoclonal anti-GFP (https://www.cellsignal.jp/products/primary-antibodies/gfp-d5-1-rabbit-mab/2956)<br>The GFP (D5.1) XP® Rabbit mAb detects GFP, YFP, and CFP-tagged proteins exogenously expressed in cells, whose antigen is perfectly conserved with the GFP tag used in this study (mClover3). The manufacturer confirmed that this antibody is available for immunohistochemistry with paraffin-embedded sections.<br>3. Anti-mouse Alexa 555 conjugated (https://www.cellsignal.jp/products/secondary-antibodies/anti-mouse-igg-h-l-f-ab-2-fragment-alexa-fluor-555-conjugate/4409)<br>4. Anti-rabbit Alexa 488 conjugated (https://www.cellsignal.jp/products/secondary-antibodies/anti-rabbit-igg-h-l-f-ab-2-fragment-alexa-fluor-488-conjugate/4412)

Eukaryotic cell lines

Policy information about cell lines and Sex and Gender in Research

Cell line source(s)	COS-7 cells
Authentication	The cell line (COS-7) was obtained from ATCC, and we checked the growth and morphology.
Mycoplasma contamination	The cell line was not tested by PCR but we checked it by hoechst staining. We did not find the mycoplasma contamination.
Commonly misidentified lines (See ICLAC register)	No commonly misidentified cell lines were not used in the study.

Animals and other research organisms

Policy information about studies involving animals; ARRIVE guidelines recommended for reporting animal research, and Sex and Gender in Research

Laboratory animals	The following fishes (Cab strain of Japanese medaka Oryzias latipes) was used for this study: wild type (113 males; 244 females), Tilling ara KO (86 males; 12 females), Tilling arb KO (78 males; 12 females), Tilling ara/arb double KO (Tilling ar DKO) (32 males; 12 females), ara KI (8 males), arb KI (8 males), TALEN ara KO (6 males), TALEN arb KO (6 males), TALEN ara/arb double KO (Talen ar DKO) (5 males). All used fishes were at the adult stage (> 4 month old after hatching). They were maintained at aquarium under artificial reproductive conditions with 14 and 10 h of light and dark cycles at 26–28˚C.<br>The OK-Cab (NBRP ID: MT830), the TILLING KO lines (ID: TA5383 for ara KO; TA3793 for arb KO) and AR-KI lines (ID: TG1341 for Ara-KI; TG1342 for Arb-KI), the TALEN KO lines (ID: Ara(del1) MT1560 for ara KO; Arb(del10) MT1561 for arb KO) are available from NBRP medaka.
Wild animals	No wild animals were used in this study.
Reporting on sex	Medaka has a male heterogametic (XX/XY) system, in which dmy/dmrt1bY on the Y chromosome determines their sexes. Therefore, In this study, we analysed the genetic sex by the amplification of dmy from the fin clips of all adult fishes. The males were used for the behavioral and morphological analyses. The females were used for the morphological analysis and fecundity test.
Field-collected samples	No field collection samples were used in this study.
Ethics oversight	Animal experiments were conducted under approval by the Institutional Animal Care and Use Committee of the National Institute for Basic Biology (15A005, 14A003, 13A023, 12A020, 11A028) and Kyushu University (A21-043-0, A19-137-0, A19-137-1, A19-137-2, A29-088-0, A29-088-1, A29-088-2).

Note that full information on the approval of the study protocol must also be provided in the manuscript.
